# VEGF/Flk1 Mechanism is Involved in Roxarsone Promotion of Rat Endothelial Cell Growth and B16F10 Xenograft Tumor Angiogenesis

**DOI:** 10.1038/s41598-019-53870-3

**Published:** 2019-11-22

**Authors:** Shihao Chen, Jinge Xu, Qianhan Wei, Zeting Zhao, Xin Chen, Hengmi Cui, Yumei Zhang

**Affiliations:** 1 College of Veterinary Medicine, Yangzhou University, Yangzhou, Jiangsu China; 2 College of Animal Science and Technology, Yangzhou University, Yangzhou, Jiangsu China; 3 Guizhou Animal Husbandry and Veterinary Research Institute, Guiyang, Guizhou China; 4 Jiangsu Co-innovation Center for Prevention and Control of Important Animal Infectious Diseases and Zoonoses, Yangzhou, Jiangsu China

**Keywords:** Mechanism of action, Growth factor signalling

## Abstract

The potential angiogenic effect of roxarsone, a feed additive widely used to promote animal growth worldwide, was demonstrated recently. We explored the mechanism of vascular endothelial growth factor (VEGF) and its receptor (VEGFR) in roxarsone promotion of rat vascular endothelial cells (ECs) and B16F10 mouse xenografts. ECs were treated with 0.1–50 μM roxarsone or with roxarsone plus 10 ng/mL VEGF, VEGFR1 (Flt1), or VEGFR2 (Flk1) antibodies for 12–48 h to examine their role in cell growth promotion. Small interfering RNA (siRNA) targeting *Vegf*, *Flt1*, and *Flk1* were transfected in the ECs, and we measured the expression level, cell proliferation, migration, and tube formation ability. The siRNA targeting *Vegf* or *Flk1* were injected intratumorally in the B16F10 xenografts of mice that received 25 mg/kg roxarsone orally. Cell viability and VEGF expression following roxarsone treatment were significantly higher than that of the control (*P* < 0.05), peaking following treatment with 1.0 μM roxarsone. Compared to roxarsone alone, the VEGF antibody decreased cell promotion by roxarsone (*P* < 0.05), and the Flk1 antibody greatly reduced cell viability compared to the Flt1 antibody (*P* < 0.01). Roxarsone and Flk1 antibody co-treatment increased supernatant VEGF significantly, while cellular VEGF was obviously decreased (*P* < 0.01), whereas there was no significant difference following Flt1 antibody blockade. The siRNA against *Vegf* or *Flk1* significantly attenuated the roxarsone promotion effects on EC proliferation, migration, and tube-like formation (*P* < 0.01), whereas the siRNA against *Flt1* effected no obvious differences. Furthermore, the RNA interference significantly weakened the roxarsone-induced increase in xenograft weight and volume, and VEGF and Flk1 expression. Roxarsone promotion of rat EC growth, migration, and tube-like formation *in vitro* and of B16F10 mouse xenograft model tumor growth and angiogenesis involves a VEGF/Flk1 mechanism.

## Introduction

Angiogenesis, which involves endothelial cell (EC) proliferation and migration and the formation of new capillaries, is a critical homeostatic mechanism in organs and tissues^1^. Many proangiogenic factors such as growth factors, cytokines, lipid metabolites, and cryptic fragments of hemostatic proteins regulate angiogenesis. Vascular endothelial growth factor (VEGF)-A is notable as the most potent promoter of angiogenesis; it binds to the VEGF receptors (VEGFR) VEGFR1 (Flt1) and VEGFR2 (Flk1), playing a key role in adult vasculature development and maintenance^2,3^. Flk1 appears to mediate almost all of the known cellular responses to VEGF^4^, while the function of Flt1 is less well-defined, although it is thought to modulate Flk1 signaling^5–7^.

Physiological and pathological factors can induce VEGF release^8,9^. VEGF overexpression is more closely related with many ischemic disease and benefits tumor growth^10,11^. Numerous studies have implicated that VEGF indicates poor prognosis in some cancers, where overall survival and disease-free survival are decreased in tumors overexpressing VEGF^12^.

Roxarsone (4-hydroxy-3-nitro-benzenearsonic acid) is widely used in many countries as a feed additive in animal production to enhance weight gain, improve feed efficiency, and control intestinal parasites^13,14^. It can be excreted almost as its parent drug in manure^15–17^; easily leaches from poultry litter^18–21^; and enhances arsenic levels in open water or underground water, soil, or plants in the local area^22–25^. Consequently, this may lead to environmental pollution and food chain contamination^26^. As a precaution, the environmental risk of using roxarsone as a feed additive warrants further evaluation^27^. Little is known about the potential human health effects from roxarsone released into the environment from poultry litter or from residual compounds in animal products.

Roxarsone promotes the angiogenic phenotype in human ECs^28^ and promotes rat EC growth and VEGF expression^29^ *in vitro*. We have previously reported the proangiogenic and tumor-promoting effects of roxarsone *in vivo*^30^.

In the present study, we investigated whether roxarsone promotes rat EC growth via VEGF/Flk1 by using targeted small interfering RNA (siRNA) interference of *Vegf* (siVEGF) and its receptor *Flt1* or *Flk1* (VEGFR2) genes (siFlt1 and siFlk1, respectively), or by antibody blockade of the related signal molecule in cell models of proliferation, migration, and tube formation. We found that inhibiting VEGF and VEGFR2 (Flk1) attenuated roxarsone-induced proliferation, migration, and tube formation in rat ECs. RNA interference of the *Vegf* and *Flk1* genes attenuated mouse B16F10 xenograft growth and tumor angiogenesis. Our results indicate that VEGF/Flk1 signaling is involved in roxarsone-induced promotion in rat ECs growth and B16F10 mouse xenografts.

## Results

### Roxarsone promoted EC growth and VEGF expression

**Figure 1:**
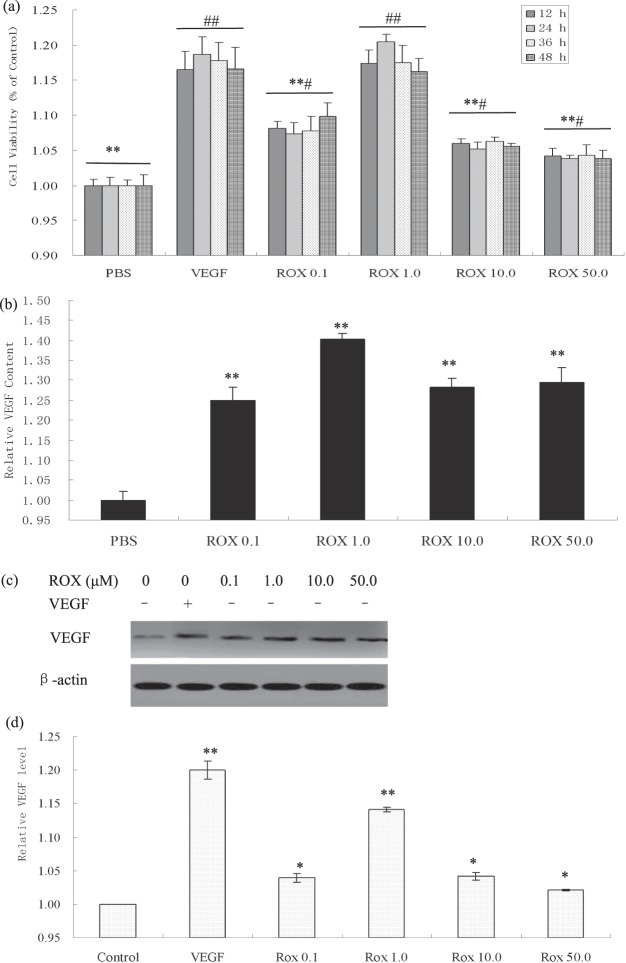
Effects of roxarsone (ROX) on viability and VEGF/VEGFR expression in EC cultures. (**a**) MTT assay determination of the viability of ECs treated with PBS (NC), 0.1–50.0 μM roxarsone, or 10 ng/mL VEGF (positive control). Results are the mean ± SEM of the OD value of triplicate samples relative to the PBS control and are representative of three independent experiments. **P < 0.01 relative to 10 ng/mL VEGF; ^#^*P* < 0.05, ^##^*P* < 0.01 relative to PBS. (**b**) ELISA of VEGF contents in supernatants of cells treated with 0.1–50.0 μM roxarsone or 10 ng/mL VEGF for 24-h. **P < 0.01 relative to PBS. (**c**) Western blotting of VEGF in total cell lysates; β-actin were used as the loading control. (**d**) Quantitative analysis of VEGF, Flt1, and Flk1 levels in cell lysates. Values are the mean ± SEM of VEGF expression standardized to β-actin expression in three independent experiments. *P < 0.05, **P < 0.01 relative to PBS by analysis of variance (ANOVA).

Following 12 h, 24 h, 36 h, and 48 h exposure, the ECs treated with roxarsone and 10 ng/mL VEGF (positive control) had significantly higher relative viability and VEGF expression than the PBS-treated negative control (*P* < 0.05, Fig. 1a). The respective treatment groups had similar cell growth trends throughout the treatment period. Roxarsone appeared to have a bilateral promoting effect on cell proliferation: 0.1–1.0 μM roxarsone increased viability and 10–50 μM roxarsone decreased promotion. However, all OD ratio values demonstrated that 0.01–50 μM roxarsone promoted proliferation in the rat EC cultures.

Both the supernatant (Fig. 1b) and lysate (Fig. 1c,d) of all treatment groups had significantly higher VEGF expression than the PBS control (*P* < 0.05). VEGF expression peaked following 1.0 μM roxarsone treatment. These results demonstrate that roxarsone induces EC VEGF expression and secretion. Previously, we demonstrated that roxarsone promotes EC growth and VEGF expression^29,31^, which were similar to the findings of others^28^.

Additionally, 1.0 μM roxarsone could promote EC proliferation, migration, and tube-like formation.

### VEGF/VEGFR blockade attenuated the roxarsone promotion effects on ECs Effect of roxarsone on viability following VEGF/VEGFR blockade

**Figure 2:**
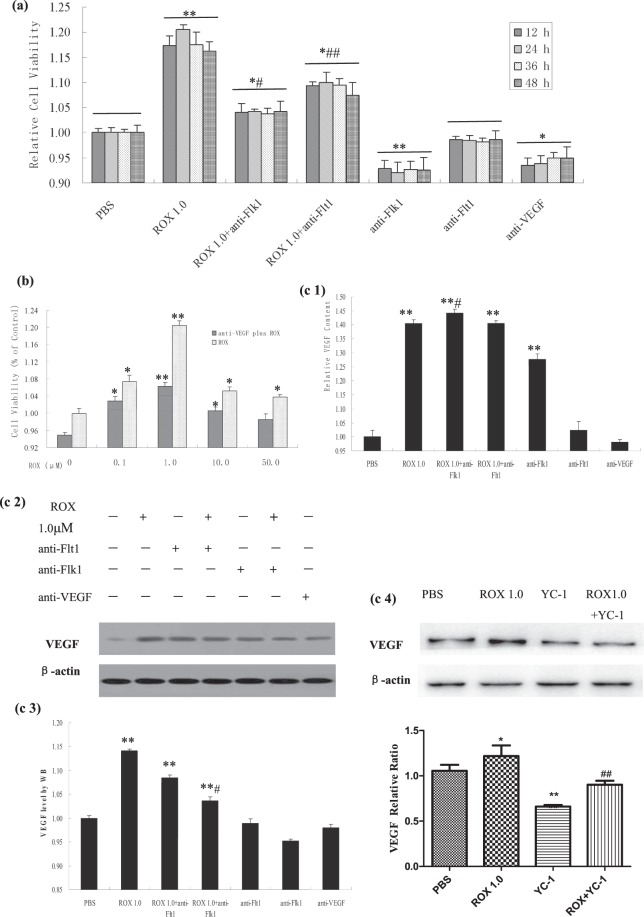
The effect of ROX plus at-VEGF/at-VEGFR on viability and VEGF expression in EC cultures. MTT assay of rat aortic following 12–48-h treatment with ROX and/or at-VEGF or at-VEGFR. (**a**) MTT assay determination of the viability of cells treated for 12–48 h with 1.0 μM ROX and/or at-VEGFR or 10 ng/mL at-VEGF. (**b**) MTT assay determination of the viability of cells treated with PBS or 0.1–50.0 μM ROX and/or 10 ng/mL at-VEGF for 24 h. (**c1**) ELISA of culture supernatants from EC treated with 1.0 μM ROX and/or 10 ng/mL at-VEGF/at-VEGFR. (**c2**) Western blotting of total cell lysates from ECs treated with 1.0 μM ROX and/or 10 ng/mL at-VEGF/at-VEGFR. β-Actin was used as the loading control. (**c3**) Quantitative analysis of VEGF levels in cell lysates. (**c4**) Western blotting of total cell lysates from ECs treated with 1.0 μM ROX and/or 50 μM YC-1. Values are the mean ± SEM of VEGF expression standardized to β-actin expression in three independent experiments. Results are the mean ± SEM of the OD value of triplicate samples relative to the PBS control and are representative of three independent experiments. *P < 0.05, **P < 0.01 relative to *P*BS; ^#^*P* < 0.05, ^##^*P* < 0.01 relative to 1.0 μM ROX by ANOVA.

To explore whether VEGF was involved in roxarsone promotion of rat EC growth under VEGF antibody blockade, MTT assay was performed on EC cultures following 24-h treatment with 0.1–50 μM roxarsone plus 10 ng/mL anti-VEGF (Fig. 2a). With the exception of 50 μM roxarsone plus anti-VEGF, roxarsone plus anti-VEGF significantly decreased cell viability compared to roxarsone alone (*P* < 0.05), where 1.0 μM roxarsone plus anti-VEGF induced a maximal decrease (*P* < 0.01).

To study the effect of VEGFR on roxarsone promotion of rat EC proliferation following VEGFR1 or VEGFR2 blockade, MTT assay was performed on cell cultures following 12–48-h incubation with 1.0 μM roxarsone or 1.0 μM roxarsone plus 10 ng/mL anti-Flt1 or anti-Flk1 (Fig. 2b). The 1.0 μM roxarsone and 1.0 μM roxarsone plus anti-VEGFR groups had significantly higher relative OD values than the PBS control (*P* < 0.05). However, the roxarsone plus anti-VEGFR groups both had obviously lower OD rates than the 1.0 μM roxarsone-alone group (*P* < 0.05). Anti-Flk1 antagonism of the roxarsone cell growth promotion was more obvious than that of anti-Flt1, implying that VEGFR2 may play a major role compared to VEGFR1 in roxarsone promotion of rat EC proliferation. The anti-VEGF and anti-VEGFR groups had clearly lower cell proliferation compared to the PBS group (*P* < 0.05).

### Effect of roxarsone on VEGF expression following VEGF/VEGFR blockade

To investigate whether roxarsone promotion of EC growth affected VEGF expression, we performed ELISA of the supernatant from EC cultures treated with 1.0 μM roxarsone and/or 10 ng/mL anti-VEGF/anti-VEGFR, and total cell lysates were analyzed using western blotting. The relative cellular VEGF content following treatment with 1.0 μM roxarsone, 1.0 μM roxarsone plus anti-VEGFR, and anti-Flk1 only were significantly higher than that of the PBS control (*P* < 0.01). There was no clear difference between the anti-Flt1 and control groups, although the anti-VEGF group had obviously lower VEGF expression than the control (*P* < 0.05, Fig. 2c,l). The roxarsone plus anti-Flk1 group had higher VEGF levels than the roxarsone-alone group (*P* < 0.05), whereas there was no clear difference between the roxarsone plus anti-Flt1 and roxarsone-alone groups (*P* > 0.05). Interestingly, the anti-Flk1 group had significantly higher VEGF levels than the control (*P* < 0.05), indicating that VEGFR2 blockade may stimulate VEGF expression by ECs. Fig. 2c2,c3 show that the roxarsone-alone, roxarsone plus anti-VEGFR, and anti-Flk1–alone groups had significantly higher lysate VEGF levels than the PBS control (*P* < 0.01), but there was no clear difference between the at-Flt1 or anti-VEGF–alone group compared to the control (*P* > 0.05). Compared to roxarsone alone, the roxarsone plus anti-Flk1 group had much lower VEGF expression (*P* < 0.01), whereas there was no obvious difference in the roxarsone plus anti-Flt1 group (*P* > 0.05). In addition, ECs were co-treated with roxarsone and the hypoxia-inducible factor (HIF)-1α inhibitor YC-1 to explore the mechanism of roxarsone-induced VEGF promotion. Fig. 2c4 shows that 50 μM YC-1 significantly decreased the VEGF levels as compared with that of the PBS group. The VEGF content in the 1.0 μM roxarsone plus 50 μM YC-1 group was much lower than that in the 1.0 μM roxarsone-alone group, indicating that YC-1 weakened the roxarsone-induced upregulation of VEGF significantly.

### Vegf/Vegfr silencing attenuated the roxarsone promotion effects on Ecs siVEGF/siFlk1 attenuated the effects of roxarsone on proliferation and migration

**Figure 3:**
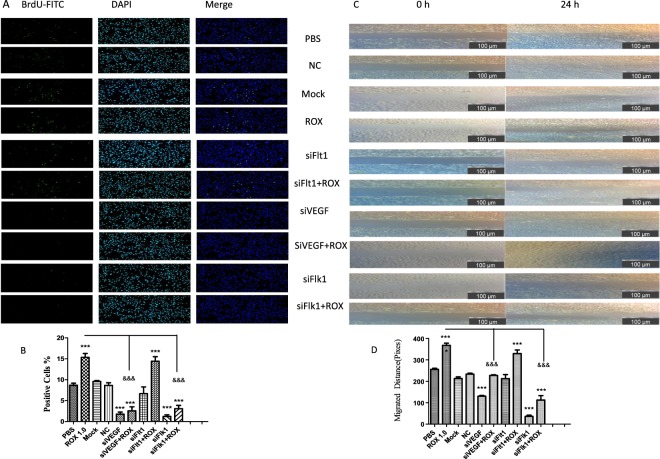
Effect of ROX plus siVEGF/siVEGFR on EC proliferation and migration. (**a**) BrdU staining of ECs treated with the indicated reagents for 24 h (×200 magnification). Representative images are shown. (**b**) The number of BrdU-positive cells presented as the mean ± SEM of three independent experiments. (**c**) Rat EC monolayers were wounded with a pipette and treated with the indicated reagents for 24 h. Representative images (×100 magnification) are shown. (**d**) The distance the cells had migrated. The results are the mean ± SEM of three independent experiments. ***P < 0.001 vs. PBS control; ^&&&^*P* < 0.001 vs. 1.0 μM ROX.

The effect of roxarsone or siVEGF/siFlk1/siFlt1 on EC proliferation was determined by BrdU staining (Fig. 3a,b). The roxarsone groups had much higher numbers of BrdU-positive cells than the control (*P* < 0.001), while the siVEGF or siFlk1 groups had much fewer BrdU-positive cells than the control (*P* < 0.001), but there was no difference between the siFlt1 group and the control (*P* > 0.05). The 1.0 μM roxarsone plus siVEGF or siFlk1 group had extremely significantly fewer BrdU-positive cells compared to the 1.0 μM roxarsone group (*P* < 0.001); there was no clear difference between the 1.0 μM roxarsone plus siFlt1 group and the 1.0 μM roxarsone group (*P* > 0.05).

The effect of roxarsone or siVEGF/siFlk1/siFlt1 on EC migration was determined by the scratch assay (Fig. 3c,d). The numbers of ECs that migrated into the blank section of the scratch differed between the treatments. Compared to the control, the 1.0 μM roxarsone group had extremely significantly increased EC migration (*P* < 0.001), whereas the siVEGF and siFlk1 groups had extremely significantly decreased EC migration (*P* < 0.001); the siFlt1 group was not significantly different (*P* > 0.05). EC migration in the Mock (transfection reagent) and NC siRNA groups did not differ from that of the control. The 1.0 μM roxarsone plus siVEGF or siFlk1 groups had clearly significantly decreased EC migration compared to the 1.0 μM roxarsone group (*P* < 0.001). The results demonstrate that, instead of VEGFR1 (Flt1), VEGF and VEGFR2 (Flk1) play an important role in roxarsone promotion of EC proliferation and migration.

### siVEGF/siFlk1 attenuated the effect of roxarsone on tube formation

**Figure 4:**
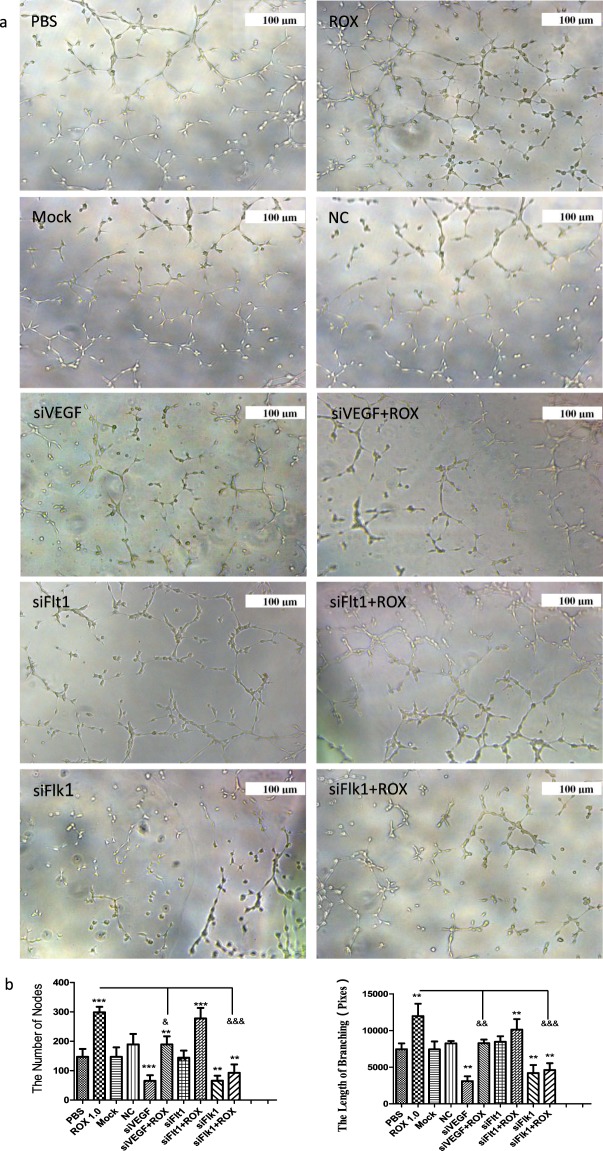
The effects of ROX plus siVEGF/siVEGFR on EC tube formation. (**a**) Representative images of tubular structures in each treatment group, scale bar = 100 μm. (**b**) Node numbers and branch lengths in five randomly selected images from each group. The results are the mean ± SEM of three independent experiments. **P < 0.01, ***P < 0.001 vs. PBS control; ^&&^*P* < 0.01, ^&&&^*P* < 0.001 vs. 1.0 μM ROX.

Figure 4a shows photographs of the capillary-like tubes that formed in the Matrigel tube formation assay. Compared to the PBS control, the node numbers and branch lengths (Fig. 4b) in the 1.0 μM roxarsone group were extremely significantly increased (*P* < 0.001), whereas that in the siVEGF and siFlk1 groups were extremely significantly decreased (*P* < 0.001); the siFlt1 group was not significantly different (*P* > 0.05). There were no clear differences between the Mock and NC siRNA groups with the PBS control (*P* > 0.05). The 1.0 μM roxarsone plus siVEGF or siFlk1 groups had clearly significantly decreased node numbers and branch lengths compared to the 1.0 μM roxarsone group (*P* < 0.001). The data prove that, instead of VEGFR1 (Flt1), VEGF and VEGFR2 (Flk1) play an important role in roxarsone promotion of tube formation in ECs *in vitro*.

### siVEGF/siFlk1 attenuated the effect of roxarsone on VEGF/VEGFR levels

**Figure 5:**
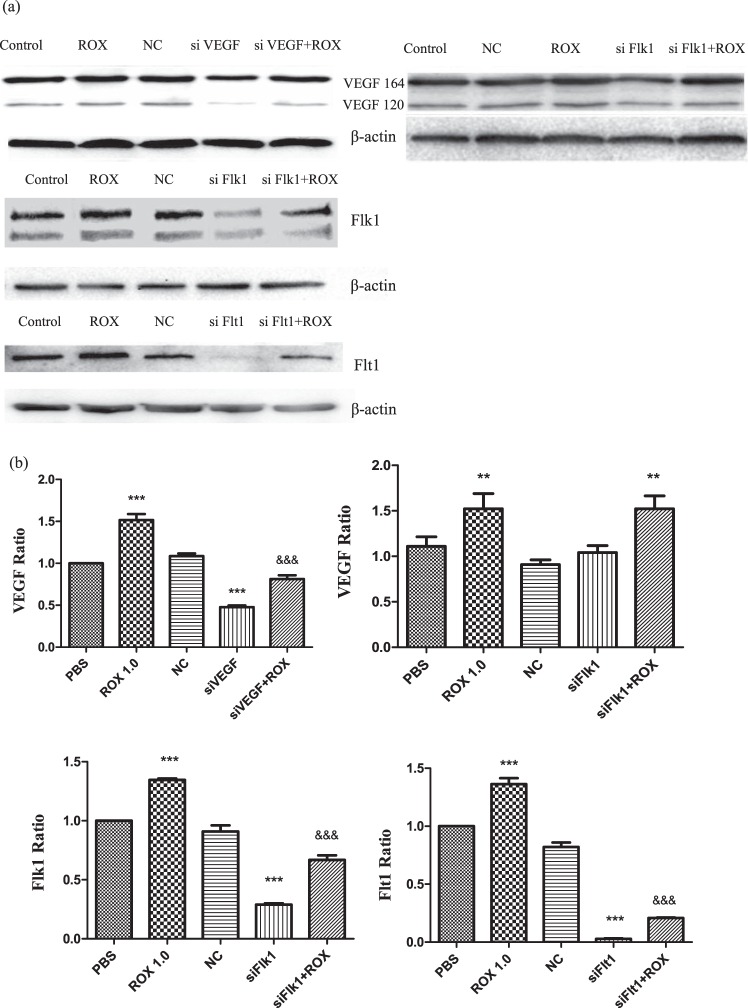
Effect of ROX plus siVEGF/siVEGFR on EC VEGF/VEGFR expression. (**a**) Western blots of VEGF, Flk1, and Flt1 in total cell lysates from ECs that had been incubated with the indicated reagents for 24 h; β-actin was used as a loading control. (**b**) Standardization of β-actin expression for determining the total VEGF, Flk1, and Flt1 levels in the cell lysates. The results are the mean ± SEM of three independent experiments. ***P < 0.001 vs. PBS control; ^&&&^*P* < 0.001 vs. 1.0 μM ROX.

Figure 5 shows the western blot analysis of VEGF, Flk1, and Flt1 levels in ECs after the 24-h treatment. siVEGF clearly attenuated total VEGF expression (VEGF164 and VEGF120 of rat VEGF isoforms were detected in the assay); siFlk did not appear to affect VEGF expression (Fig. 5a). The siRNAs decreased Flk1 and Flt1 expression. Compared to the PBS control, the 1.0 μM roxarsone group had extremely significantly increased VEGF, Flk1, and Flt1 levels, with a relative ratio of about 1.5, 1.4, and 1.4, respectively (*P* < 0.001). VEGF, Flk1, and Flt1 protein levels in the siVEGF, siFlk1, and siFlt1 groups, respectively, were extremely significantly decreased (*P* < 0.001), whereas there was no significant difference in the NC siRNA groups (*P* > 0.05). The siVEGF plus 1.0 μM roxarsone, siFlk1 plus 1.0 μM roxarsone, and siFlt1 plus 1.0 μM roxarsone groups all had extremely significantly lower VEGF, Flk1, and Flt1 levels, respectively, than the 1.0 μM roxarsone group (*P* < 0.001), see Fig. 5b.

### siVEGF/siFlk1 attenuated the effect of roxarsone on B16F10 xenograft growth

**Figure 6:**
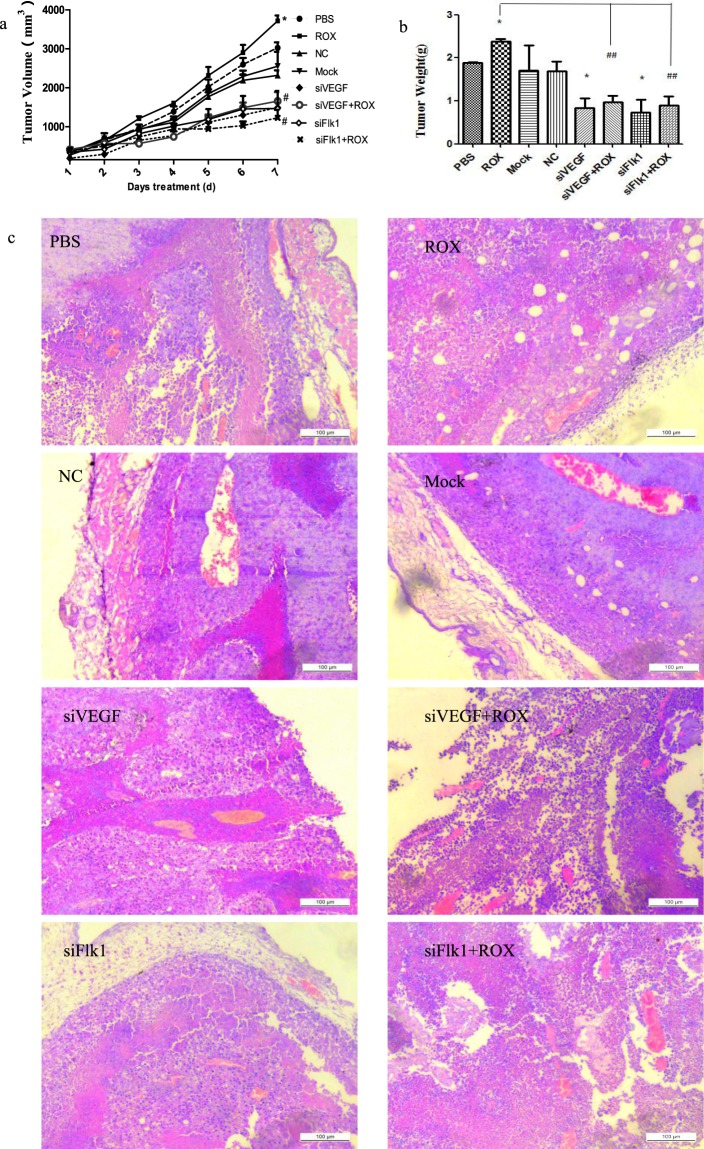
Effect of ROX plus siVEGF/siFlk1 on B16F10 xenograft growth. The tumor volume (**a**) and weight (**b**) of the B16F10 xenografts at 7 days of treatment. *P < 0.05 vs. PBS control; ^#^*P* < 0.05, ^##^*P* < 0.01 vs. 5 mg/kg ROX. (**c**) HE staining analysis of B16F10 tumor tissue; scale bar = 100 μm.

Figure 6 shows the effect of roxarsone and siVEGF/siFlk1 on B16F10 xenograft growth. The 25 mg/kg roxarsone group (Fig. 6a) had larger tumor volumes than the PBS control at day 3–7 of treatment, with a significant difference at day 7 of treatment (*P* < 0.05); the siVEGF, siFlk1 and roxarsone plus siVEGF or siFlk1 groups all had lower tumor volumes compared to the control, with a significant difference at day 7 of treatment (*P* < 0.05). Compared to the roxarsone group, the roxarsone plus siVEGF or siFlk1 group appeared to have lower-volume tumors (*P* < 0.01). There was no significant difference in tumor volume between the NC and Mock groups and the control. The tumor weights (Fig. 6b) followed the same trends as the tumor volumes. Roxarsone alone increased tumor weight, while roxarsone plus siVEGF or siFlk1 decreased tumor weight significantly (*P* < 0.01).

Figure 6c shows the HE staining analysis of the B16F10 tumor tissue sections; the tumor cell growth patterns and blood vessel sizes varied between the treatments. The xenograft tumors in the PBS, 25 mg/kg roxarsone, NC, and Mock groups appeared to have more integrated and tighter structure and organization compared to the siRNA-alone or roxarsone plus siRNA groups, and the cells showed inward cluster growth centered on a blood vessel. The roxarsone plus siVEGF or siFlk1 groups had clearly greater tumor cell density than the siRNA-alone groups.

### CD34 immunohistochemistry and VEGF and Flk1 Western Blot Analysis in B16F10 xenografts

**Figure 7:**
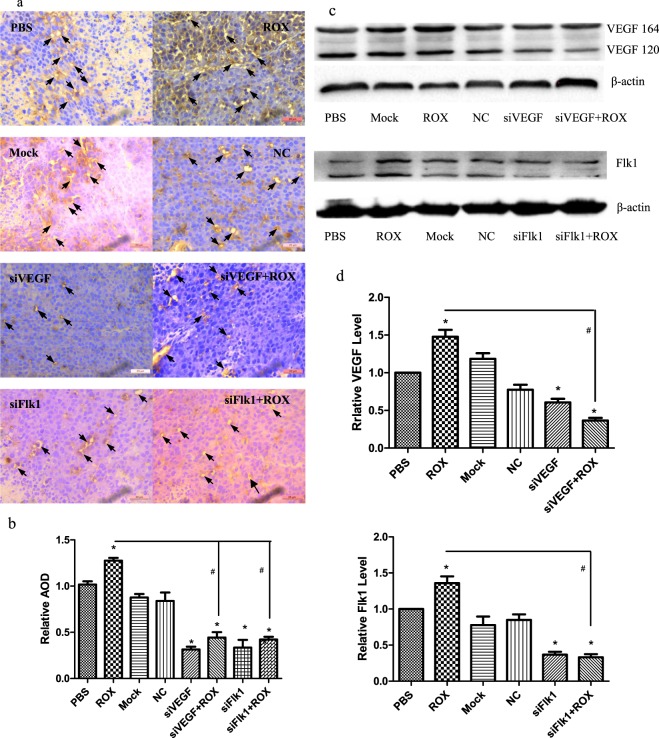
Effect of ROX plus siVEGF/siFlk1 on B16F10 xenograft CD34 and VEGF/Flk1 expression. (**a**) CD34 immunohistochemical analysis of paraffin-embedded tumor slices; arrows indicate CD34 positive expression; scale bar = 20 μm. (**b**) The AOD of B16F10 xenograft CD34-positive staining under five random visual fields from each mouse was analyzed statistically using ImageJ. (**c**) Western blots of VEGF and Flk1 in B16F10 xenografts; β-actin was used as a loading control. (**d**) Standardization of β-actin expression for determining the total VEGF and Flk1 levels of the tumor tissue. The results are the mean ± SEM of three independent experiments. *P < 0.05 vs. PBS control; ^#^*P* < 0.05 vs. 1.0 μM ROX.

CD34 antigens such as CD31 or CD146 are important markers of vascular-derived progenitor cells and ECs^32^. VEGF and VEGFR regulate EC growth and migration, and angiogenesis^33^. The CD34 immunohistochemical staining, average optical density (AOD) statistics by ImageJ software (Fig. 7a,b), and western blot analysis of VEGF and Flk1 (Fig. 7c,d) in the B16F10 xenografts indicated that roxarsone could promote tumor angiogenesis, an important process in the development of most tumors, and might further explain the co-carcinogenicity of roxarsone. siVEGF and siFlk1 decreased the CD34, VEGF, and Flk1 expression promoted by roxarsone; the roxarsone plus siRNA groups had significantly lower VEGF and Flk1 levels than the roxarsone-alone group (*P* < 0.05). The findings demonstrate that RNA interference of *Vegf* and *Flk1* weakens roxarsone-promoted tumor angiogenesis in the B16F10 xenograft mouse model.

## Discussion

VEGF, a highly conserved genetic pathway that has evolved from simple to complex systems, is critical to vascular development and angiogenesis^34^. In the present study, 10 ng/mL VEGF or 0.1–50 μM roxarsone significantly promoted rat EC growth, with 1.0 μM roxarsone and 10 ng/mL VEGF inducing similar growth-promoting effects. The promotion effect on rat EC growth was in accordance with the promotion of the angiogenic phenotype in human ECs (human aortic ECs and lung microvascular ECs) by 0.01–10 μM roxarsone^28^. Moreover, 10 ng/mL VEGF or 0.1–50 μM roxarsone increased VEGF expression in both the culture supernatants and cell lysates, similar to what we have reported previously^29^. We also checked the HIF-1α signals upstream of VEGF: RT-PCR and western blotting showed that HIF-1α levels were significantly increased after 0.1, 1.0, and 10.0 μM roxarsone treatment (Figure S). The HIF-1α inhibitor YC-1 decreased the roxarsone-increased VEGF levels, and also decreased the EC activity. HIF-1α is one of the best-characterized stimuli for angiogenic response, and *Vegf* is one of its target genes. VEGF expression is mediated by HIF-1α, which, combined with the promoter region of the *Vegf* gene, induces VEGF expression^35^. Accordingly, based on the present study findings, it is reasonable that roxarsone promotes rat ECs via the HIF-1α/VEGF pathway. We demonstrate that VEGF signaling is involved in roxarsone promotion of rat EC proliferation *in vitro*. The promoter effect of roxarsone on angiogenic vessels has been reported in a Matrigel plug assay and chicken embryo allantoic membrane (CAM) model *in vivo*^30^. The angiogenesis-promoting effect of roxarsone has also been found in microvessel outgrowth from *ex vivo* rat aorta ring cultures^29^. Further investigation of roxarsone on quiescent vessel are needed. In the present study, there was no obvious difference in either angiogenic vessels or quiescent vessels. Angiogenesis is mainly characterized by the protrusion and outgrowth of capillary buds and sprouts from pre-existing blood vessels. Angiogenesis or neovascular formation are associated with many vascular diseases, such as fundus vascular hyperplasia and various solid tumors. Roxarsone used in animal production induces the risk of vascular disease because of its angiogenesis promotion.

VEGFA binding to VEGFR on ECs is a prerequisite for VEGF regulation, which initiates various downstream signaling cascades and promotes vessel permeability and EC proliferation and migration, and finally results in the formation of mature blood vessels^36,37^. VEGFR contains an extracellular VEGF-binding domain comprising seven immunoglobulin-like domains, a single transmembrane region, and a cytoplasmic tyrosine kinase domain^6,38,39^. VEGFR2 largely mediates VEGFA-induced proangiogenic signaling, whereas the function of VEGFR1 is unclear. VEGFR1 is likely a decoy receptor that sequesters VEGFA from VEGFR2^40,41^, while VEGFR2 is required for EC migration and proliferation during angiogenesis^42^. In the present study, roxarsone promoted rat EC viability, proliferation, migration, and tube formation with the synchronous increase of the expression of VEGF and its receptors Flt1 or Flk1. However, VEGF, Flt1, and Flk1 appear to have different effects on rat EC functions. Compared to roxarsone, VEGF and Flk1 blockade decreased cell viability by almost 50%, while Flt1 blockade decreased cell viability by almost one-third (Fig. 2). Roxarsone plus *Vegf* silencing decreased EC proliferation by about four times, and decreased EC migration and VEGF expression by one-third compared to 1.0 μM roxarsone alone (Figs. 3b,d and 4b).

Anti-Flk1 blockade significantly increased the VEGF content of the supernatant, but the VEGF levels in the rat ECs decreased (no significant difference); cell viability was halved. In contrast to the effect of 1.0 μM roxarsone alone, roxarsone plus *Flk1* silencing decreased cell proliferation by about four times, migration by two-thirds, and tube formation by 50%.

Compared to the PBS control, Flt1 blockade did not significantly alter cell viability, VEGF secretion in culture medium, and VEGF levels in the ECs. The cell viability in the 1.0 μM roxarsone plus at-Flt1 group was decreased by about one-third compared to that in the 1.0 μM roxarsone group. *Flt1* silencing decreased Flt1 expression significantly, but EC proliferation, migration, and tube formation in the roxarsone plus *Flt1*-silenced group were not significantly different from that in the 1.0 μM roxarsone-alone group. Although roxarsone increased VEGF and Flk1 or Flt1 expression, Flt1 did not appear to affect EC function. These data are concordant with that in several reports stating that VEGFR1 negatively regulates VEGFR2 and suppresses excessive VEGFR2 signaling for vascular balance. Crosstalk with VEGFR2 might influence the biological outcome of stimulating or inhibiting VEGFR1 activity^43^.

In our experiment, blocking VEGFR2 in cell cultures co-treated with roxarsone had clearly stronger effects on cell functions such as viability, proliferation, migration, and tube formation than that of VEGFR1 blockade, although roxarsone induced VEGF, Flt1, and Flk1 expression. VEGF concentrations in the culture supernatant and VEGF levels in the cells exposed to roxarsone and VEGFR1 blockade were not significantly altered compared to that in cells exposed to roxarsone alone (*P* > 0.05). Evidently, VEGFR1 does not predominate in VEGF signaling in roxarsone promotion of rat EC proliferation, migration, and capillary-like structure formation. The VEGFR1 blockade did not weaken the roxarsone-induced VEGF effects; a study on VEGFA–induced tube formation by human microvascular ECs^44^ and in VEGFR1 knockout mice^45^ reported similar results. There was a clear decrease in VEGF expression in cells co-treated with anti-Flk1 and roxarsone compared to cells treated with roxarsone alone (*P* < 0.01). The VEGF concentration in the culture supernatant of cells co-treated with anti-Flk1 and roxarsone may have been increased because VEGFR2 was blocked in the cell membrane and because there was insufficient binding of the roxarsone-promoted VEGF secretion to the membrane, resulting in VEGF accumulating in the cell cultures due to the lack of VEGFR2 binding. Interaction between VEGFR2, endothelial nitric oxide synthase, and other signaling molecules regulates VEGF-induced EC proliferation^46,47^. In a future study, we intend to study the VEGF signaling pathway and cross-interactions with other signal molecules in roxarsone promotion of EC proliferation.

In addition, we used different animal models in the present study. The promoter effect of roxarsone on the growth, migration, and tube-like formation of rat vascular ECs was investigated by MTT assay, scratch assay, and tube formation assay *in vitro*, and the promoter effect on angiogenesis was examined using a B16F10 xenograft mouse model *in vivo*. Angiogenesis plays a leading role in tumorigenesis and tumor development. Typically, tumor xenograft models of nude mice or other mice such as C57BL/6 mice are used. Previously, we used the B16F10 xenograft model in C57BL/6 mice^31^ and MCF-7 nude mice^30^, and the B16F10 C57BL/6 mouse model was relatively easy to establish and cheap. We also believe that other tumor models may be suited to the work; accordingly, we will focus next on the selective effect of roxarsone on different tumors.

## Conclusions

We demonstrate that VEGF signaling plays an important role in roxarsone promotion in rat ECs. Roxarsone promotion of rat EC growth, migration, and tube-like formation *in vitro* and of B16F10 mouse xenograft model tumor growth and angiogenesis involves a VEGF/Flk1 mechanism.

## Methods

### Ethical approval of the study protocol

All protocols were reviewed by the Committee for the Ethics of Animal Experiments of Yangzhou University (Yangzhou, China). The experiments were carried out in accordance with the Regulations for the Administration of Affairs Concerning Experimental Animals in China and EU Directive 2010/63/EU for animal experiments.

### Chemicals and experimental animals

Roxarsone, sodium heparin, and trypsin were purchased from Sigma-Aldrich (St. Louis, MO, USA). Roxarsone stock solution (1.0 mM) was prepared, dissolved in about 5 mL methanol, and then diluted to 50 mL with deionized water. The solution (0.1–50.0 μM) was further diluted with incubation medium. Recombinant VEGF165 was purchased from Lonza (Basel, Switzerland). Dulbecco’s modified Eagle’s medium (DMEM), penicillin/streptomycin, and fetal bovine serum (FBS) were purchased from Gibco (Carlsbad, CA, USA). Rabbit polyclonal antibodies against VEGFA (ab46154, anti-VEGF), Flk1 (ab11939, anti-Flk1), and Flt1 (ab2350, anti-Flt1) were purchased from Abcam (Cambridge, MA, USA); anti–β-actin antibody was purchased from Santa Cruz Biotechnology (Santa Cruz, CA, USA); anti-rabbit horseradish peroxidase–conjugated immunoglobulin G (IgG-HRP) was from Pierce (Rockford, IL, USA). YC-1 was from Selleck Chemicals (Houston, TX, USA). Tissue culture plates or flasks were from Falcon (Franklin Lakes, NJ, USA). Entranster-R4000 and Entranster-*in vivo* transfection reagent were from Engreen Biosystem Co., Ltd. (Beijing, China).

All siRNAs purified by high-performance liquid chromatography were chemically synthesized by Suzhou GenePharma Co., Ltd. (Suzhou, China). Pure siRNAs were designated siVEGF targeting rat *Vegfa* gene (NCBI ID 83785) (sense, GGGCUGCUGCAAUGAUGAATT; anti-sense, UUCAUCAUUGCAGCAGCCCTT); siFlt1 targeting rat *Flt1* (sense, CCCGGGAUAUUUAUAAGAATT; anti-sense, UUCUUAUAAAUAUCCCGGGTT); siFlk1 targeting rat *Flk1* (sense, CCGAAUCCCUGUGAAGUAUTT; anti-sense, AUACUUCACAGGGAUUCGGTT), while the negative control (NC: sense, UUCUCCGAACGUGUCACGUTT; anti-sense, ACGUGACACGUUCGGAGAATT) did not target any rat gene.

Male C57BL/6 mice (20 ± 2 g) and female Wistar rats (200–250 g) were purchased from the Center of Comparative Medicine, Yangzhou University, Yangzhou, China. The animals were housed at 22 °C in 55% ± 5% humidity on a 12-h light/dark cycle. Food and water were provided *ad libitum*. The Yangzhou University Committee on the Ethics of Animal Experiments reviewed all protocols, which were carried out in accordance with EU Directive 2010/63/EU for animal experiments.

### Endothelial cell culture

The Wistar rats were anesthetized with 2% thiopental sodium and then humanely euthanized. EC isolation from the thoracic aorta was performed as described by Zhu *et al*.^29^, and the cells were cultured in DMEM Supplemented with 15% (v/v) FBS, L-glutamine (2 mM), 100 μg/mL sodium heparin, 4 ng/mL VEGF, and 100 U penicillin/streptomycin at 37 °C in 5% CO_2_. Once they had formed a monolayer, the ECs were subcultured in DMEM with 10% FBS, digested with 2% trypsin, and collected.

### [4, 5-dimethylthiazol-2-yl]-2, 5 diphenyl tetrazolium bromide (MTT) cell survival assay

The cells (2 × 10^3^ per well) were plated in 96-well plates and incubated overnight in reduced-serum and growth factor–containing DMEM (1:5 dilution of complete DMEM with non-supplemented DMEM). The cells were subsequently treated for 12–48 h with phosphate-buffered saline (PBS); 5 ng/mL VEGF; roxarsone (0.1, 1.0, 10.0, or 50.0 μM); or 1.0 μM roxarsone plus 10 ng/mL anti-VEGF, anti-Flt1, or anti-Flt1. After incubation, MTT reduction assays were performed at a final concentration of 0.4 mg/mL in medium. After 4 h, reduced formazan was solubilized with 150 μL dimethyl sulfoxide by shaking for 10 min, and the absorbance was measured at 570 nm in a microplate reader (BioTek Instruments, Winooski, VT, USA). The optical density (OD) rate was calculated by dividing the OD value of the test compounds by the OD values of the PBS control to evaluate the changes in cell viability caused by the treatments. Six wells were replicated for each treatment.

### VEGF enzyme-linked immunosorbent assay (ELISA) and VEGF/VEGFR Western Blot Analysis

The EC density was adjusted to 2.5 × 10^6^ cells/mL, and the cells were incubated for 4 h in culture flasks (75 cm^3^). The cells were then incubated for 24 h in roxarsone (0.10, 1.0, 10.0, or 50.0 μM); 10 ng/mL VEGF; 1.0 μM roxarsone plus 10 ng/mL anti-VEGF, anti-Flt1, or anti-Flt1; 50 μM YC-1; or PBS in complete DMEM. The VEGF concentration in the culture supernatants of conditioned medium was measured using a rat VEGF immunoassay kit (Rat VEGF DuoSet, R&D Systems, Minneapolis, MN, USA), which recognizes the soluble isoforms VEGF120 and VEGF164 with a sensitivity of 5 pg/mL. The absorbances were measured at 450 nm in a microplate reader and the VEGF concentration was calculated using a standard curve based on the OD value at 450 nm. The relative VEGF content was measured by dividing the VEGF concentration of the treatment groups by the VEGF concentration of the PBS control.

For western blotting, the remaining cells were washed three times with PBS and lysed in lysis buffer (40 mM Tris-Cl, 10 mM EDTA, 120 mM NaCl, 0.1% NP-40 with protease inhibitor cocktail [Sigma]). The protein concentrations were determined, adjusted, and a constant protein concentration (30 μg/lane) was used. Proteins were separated by 10% sodium dodecyl sulfate polyacrylamide gel electrophoresis and transferred to a nitrocellulose membrane (Amersham Pharmacia Biotech, New York, USA) using a Bio-Rad Transblot apparatus (Bio-Rad Laboratories, Hercules, CA, USA). The membrane was blocked with 5% skim milk in PBS containing 0.1% Tween-20 for 1 h at room temperature. The proteins were then incubated overnight at 4 °C with primary anti-VEGF, anti-Flt1, anti-Flk1, or anti–β-actin antibody. Thereafter, the samples were incubated with anti-rabbit IgG HRP secondary antibody for 90 min at room temperature. Immunoreactive proteins were visualized by an enhanced chemiluminescence detection system (Bio-Rad Laboratories). The grey levels of the bands were assayed by Gel-Pro Analyzer 4 software (Bio-Rad Laboratories).

### siRNA transfection

Cells (7 × 10^4^ per well) were plated in 6-well plates and incubated for 24 h. siRNA (20 µM) was prepared with diethylpyrocarbonate-treated deionized water. siRNA (12.5 µL, 20 µM) and 2.5 µL Entranster-R4000 were each diluted with 100 µL Opti-MEM (Gibco Life Technologies, Grand Island, NY, USA) for 5 min at room temperature. The transfection mixture was mixed with the siRNA and Entranster-R4000 dilutions, incubated for 20 min at room temperature, and 200 µL of the mixtures were added to each well; the final siRNA concentration was 100 nM. The transfection solution was used as the blank control. Then, the solutions were replaced with 2 mL DMEM containing 4% FBS after the plate had been incubated for 6 h at 37 °C. After 24-h siRNA transfection, the cells were examined via the 5′-bromo-2′-deoxyuridine (BrdU), scratch, and tube formation assays described below.

### Cell proliferation BrdU assay

After 24-h siRNA transfection, the ECs were treated with and without 1.0 µM roxarsone for 24 h. Then, BrdU was added at a final concentration of 30 μg/L to the medium and incubated with the cells for 4 h. The cells were fixed with methanol containing 0.04% H_2_O_2_ for 15 min, and then incubated in 2 N hydrochloric acid at 37 °C for 1 h. Subsequently, the cells were blocked in 5% bovine serum albumin (BSA) for 20 min at room temperature, and then incubated overnight with BrdU antibody (1:200 dilution) at 4 °C. After the primary antibody had been washed off, the cells were incubated with goat anti-mouse fluorescein isothiocyanate–conjugated IgG (1:50 dilution) for 30 min at room temperature away from light. This was followed by excitation at 494 nm, after which BrdU-positive cells were counted under a Leica DMI400B fluorescence microscope (Leica Camera, Wetzlar, Germany) under ×200 magnification.

### Cell migration scratch assay

The ECs were treated with and without 1.0 µM roxarsone for 24 h after 24-h siRNA transfection. The monolayer was wounded by scratching with pipette tips, and washed with PBS. The different reagents treated were added to each well, and the wells were incubated at 37 °C in a 5% CO_2_ atmosphere for 24 h. The cells were photographed at ×100 magnification under a light microscope, and the distances they had migrated were quantified by subtracting the width of the scratch at 0 h from that at 24 h, as measured by Leica QWin Pro V 3.5.1 software.

### Matrigel tube formation assay

The Matrigel assay has been widely used for *in vitro* measurement of EC tube formation and was performed as described previously^12^. Each well in 96-well plates was coated with 50 μL Matrigel, and the plates were incubated at 37 °C for 6 h to allow the Matrigel to polymerize fully. Cells collected after 24-h siRNA transfection were seeded at a density of 1.5 × 10^3^ cells per well in the prepared plates and treated with 1.0 µM roxarsone for 6 h. Images were acquired from five random fields of each well at ×200 magnification and were photographed using a Leica inverted phase-contrast microscope. Endothelial tube formation was evaluated during the incubation period. The number of closed loops and branch points were counted for representative images. Capillary-like tube formation was quantified by counting mesh numbers and branch lengths in randomly selected images from each treatment group using Image J with the Angiogenesis Analyzer plugin (Rawak Software Inc., Stuttgart, Germany).

### B16F10 xenograft mouse model and siRNA interference

B16F10 melanoma cells were gifted by the Research Group of Laboratory Animals of Yangzhou University, which had purchased them from the Institute of Biochemistry and Cell Biology, Chinese Academy of Sciences (Shanghai, China); the cells had been tested for mycoplasma contamination, and incubated in DMEM containing 10% FBS and penicillin/streptomycin. The C57BL/6 mice were housed at 22 ± 2 °C in 55% ± 5% humidity with a 12-h light/dark cycle for 1 week of acclimatization. Then, 1 × 10^5^ B16F10 cells in 0.3 mL non-supplemented DMEM were injected subcutaneously into the external surface of the right ribs of the mice. Visible tumors developed 1 week after injection. Animals with a maximum tumor length of about 0.8 cm were randomly selected, and PBS and 25 mg/kg roxarsone were administered intragastrically once daily for 1 week. Together with the roxarsone, the mixture of 33 μg siRNA (siVEGF, siFlk1, NC siRNA, or transfection control) and 15 μL Entranster-*in vivo* was injected intratumorally two times in 3 days using a 30-gauge needle. Each group had three xenograft-bearing mice. During this week, body weight and tumor length (L) and width (W) were measured using Vernier calipers once daily, and tumor volume was calculated using the formula 1/2 L × W^2^. After the animals had been sacrificed at 1 week after treatment, the tumors were excised, weighed, fixed overnight in 10% formalin, embedded in paraffin, and sectioned. The sections were stained with hematoxylin and eosin (HE) and photographed using a Leica light microscope at ×200 magnification.

The sections underwent CD34 immunohistochemical staining using routine methods. Briefly, tumor sections were deparaffinized, endogenous peroxidase was inactivated in 3% peroxide for 10 min, and antigen retrieval in 0.1 M sodium citrate was performed in a microwave oven before the sections were blocked with 5% BSA and incubated overnight at 4 °C with polyclonal antibodies against CD34 (1:200 dilution, Beyotime Biotechnology Ltd., Shanghai, China). Then, the samples were stained using a SABC-POD three-step detection kit according to the manufacturer’s instructions (Boster, Wuhan, China) and photographed using a Leica microscope at ×200 magnification.

## Supplementary information

Dataset 1

The original blots or box-plot figures

## Data Availability

The data in this manuscript are available from the corresponding author on reasonable request.

## References

[1] Risau W, Flamme I (1995). Vasculogenesis. Ann Rev Cell Dev Bio..

[2] Kiselyov A, Balakin KV, Tkachenko SE (2007). VEGF/VEGFR signalling as a target for inhibiting angiogenesis. Expert Opin Investig Drugs..

[3] Lyons JM (2010). The role of VEGF pathways in human physiologic and pathologic angiogenesis. J Surg Res..

[4] Holmes K, Roberts OL, Thomas AM, Cross MJ (2007). Vascular endothelial growth factor receptor-2: structure, function, intracellular signalling and therapeutic inhibition. Cell Signal..

[5] Karkkainen MJ, Petrova TV (2000). Vascular endothelial growth factor receptors in the regulation of angiogenesis and lymphangiogenesis. Oncogene..

[6] Roberts DM (2004). The vascular endothelial growth factor (VEGF) receptor Flt-1 (VEGFR-1) modulates Flk-1 (VEGFR-2) signaling during blood vessel formation. Am J Pathol..

[7] Elisa B, John BM, Joyce B (2011). VEGFR-1 Mediates Endothelial Differentiation and Formation of Blood Vessels in a Murine Model of Infantile Hemangioma. Am J Pathol..

[8] Dvorak HF, Brown LF, Detmar M, Dvorak AM (1995). Vascular permeability factor/vascular endothelial growth factor, microvascular hyperpermeability and angiogenesis. Am J Pathol..

[9] Rosa MC, Rocío CM, Eva F, Juan M, Miguel C (2013). Inhibition of VEGF expression in cancer cells and endothelial cell differentiation by synthetic stilbene derivatives. Bioorganic Med Chem..

[10] Ala’eddin J (2007). Vascular endothelial growth factor (VEGF), VEGF receptors expression and microvascular density in benign and malignant thyroid diseases. Int J Experim Pathol..

[11] Brodsky SV, Mendelev N, Melamed M, Ramaswamy G (2007). Vascular density and VEGF expression in hepatic lesions. J Gastrointestin Liver Dis..

[12] Shanchun G, Laronna SC, Miles F, Yuanyuan Z, Ruben RGP (2010). Vascular endothelial growth factor receptor-2 in breast cancer. Biochimica Biophysica Acta..

[13] Chapman HD, Johnson ZB (2002). Use of antibiotics and roxarsone in broiler chickens in the USA: analysis for the years 1995–2000. Poultry Sci..

[14] Nachman KE (2017). Navas-Acien A. Nitarsone, Inorganic Arsenic, and Other Arsenic Species in Turkey Meat: Exposure and Risk Assessment Based on a 2014 US Market Basket Sample. Environ Health Perspect..

[15] Jackson BP, Bertsch PM (2001). Determination of arsenic speciation in poultry wastes by IC-ICP-MS. Environ Sci Technol..

[16] Arai Y, Lanzirotti A, Sutton S, Davis JA, Sparks DL (2003). Arsenic speciation and reactivity in poultry litter. Environ Sci Technol..

[17] Garbarino JR, Bednar AJ, Rutherford DW, Beyer RS, Wershaw RL (2003). Environmental fate of roxarsone in poultry litter. I. Degradation of roxarsone during composting. Environ Sci Technol..

[18] Brown BL, Slaughter AD, Schreiber ME (2005). Controls on roxarsone transport in agricultural watersheds. Appl Geochem..

[19] Rutherford DW (2003). Environmental fate of roxarsone in poultry litter. part II. Mobility of arsenic in soils amended with poultry litter. Environ Sci Technol..

[20] Jackson BP (2003). Trance element speciation in poultry litter. J Environ Qual..

[21] Zhang YM, Chen J, Shi YJ, Liu HL (2009). Leaching of roxarsone in soil columns. J Agro-Environ Sci..

[22] Liu CW, Liu CC, Jang CS, Sheu GR, Tsui L (2009). Arsenic accumulation by rice grown in soil treated with roxarsone. J Plant Nutr Soil Sci..

[23] Yao LX (2009). Arsenic uptake by two vegetables grown in two soils amended with As-bearing animal manures. J. Hazardous Materials..

[24] Wang FM, Chen ZL, Sun YX, Gao YL, Yu JX (2006). Investigation on the pollution of organoarsenical additives to animal feed in the surroundings and farmland near hog farms. Acta Ecologica Sinica..

[25] Oyewumi O, Schreiber ME (2017). Using column experiments to examine transport of As and other trace elements released from poultry litter: Implications for trace element mobility in agricultural watersheds. Environ Poll..

[26] Nigra AE, Nachman KE, Love DC, Grau-Perez M, Navas-Acien A (2017). Poultry Consumption and Arsenic Exposure in the US Population. Environ Health Perspect..

[27] Wang CC, Wu HY, Jan TR (2010). The Effect on Animal Growth and the Impact on the Environment by Roxarsone, an Organic Arsenic Veterinary Drug. J Taiwan Veterinary..

[28] Basu P, Richik NG, Linnette EG, Linda K, Aaron B (2008). Angiogenic potential of 3-nitro-4-hydroxy benzene arsonic acid (roxarsone). Environ Health Perspect..

[29] Zhu JQ (2013). *In vitro* and *ex vivo* angiogenic effects of roxarsone on rat endothelialcells. Toxicol Lett..

[30] Zhang YM (2016). Organoarsenic Roxarsone Promotes Angiogenesis *In Vivo*. Basic Clin Pharmacol Toxicol..

[31] Wang YG (2016). Roxarsone induces angiogenesis via PI3K/Akt signaling. Cell Biosci..

[32] Fina L (1998). Expression of the CD34 gene in vascular endothelial cells. Blood..

[33] Frangiamore SJ (2018). Evaluation of Endothelial and Vascular-Derived Progenitor Cell Populations in the Proximal and Distal UCL of the Elbow: A Comparative Study. Orthop J Sports Med..

[34] Carmeliet P (2000). Mechanisms of angiogenesis and arteriogenesis. Nat Med..

[35] Semenza GL (2000). HIF-1 and human disease: one highly involved factor. Genes Dev..

[36] de Vries C (1992). The fms-like tyrosine kinase, a receptor for vascular endothelial growth factor. Sci..

[37] Waltenberger J, Claesson-Welsh L, Siegbahn A, Shibuya M, Heldin CH (1994). Different signal transduction properties of KDR and Flt1, two receptors for vascular endothelial growth factor. J Biol Chem..

[38] Ferrara N, Gerber HP, Lecouter J (2003). The biology of VEGF and its receptors. Nat Med..

[39] Takahashi H, Shibuya M (2005). The vascular endothelial growth factor (VEGF)/VEGF receptor system and its role under physiological and pathological conditions. Clin Sci (Lond)..

[40] Stuttfeld E, Ballmer-Hofer K (2009). Structure and function of VEGF receptors. IUBMB Life..

[41] Jinnin M (2008). Suppressed NFAT-dependent VEGFR1 expression and constitutive VEGFR2 signaling in infantile hemangioma. Nat Med..

[42] Rahimi N, Dayanir V, Lashkari K (2000). Receptor chimeras indicate that the vascular endothelial growth factor receptor-1 (VEGFR-1) modulates mitogenic activity of VEGFR-2 in endothelial cells. J Biol Chem..

[43] Olsson AK, Dmberg A, Kreuger J, Claesson-Welsh L (2006). VEGF receptor signalling — in control of vascular function. Mol Cell Bio..

[44] Amano H (2015). The Role of vascular endothelial growth factor receptor-1 signaling in the recovery from ischemia. PLoS One..

[45] Koolwijk P (2001). Involvement of VEGFR-2 (kdr/flk-1) but not VEGFR-1 (flt-1) in VEGF-A and VEGF-C induced tube formation by human microvascular endothelial cells in fibrin matrices *in vitro*. Angiogenesis..

[46] Jun C, Wen GJ, Asif A, Mike B (2006). Vascular endothelial growth factor-induced endothelial cell proliferation is regulated by interaction between VEGFR-2, SH-PTP1 and eNOS. Microvascular Res..

[47] Carmine G, Robin CMH, Christopher JD (2013). VEGF-mediated phosphorylation of eNOS regulates angioblast and embryonic endothelial cell proliferation. Dev Bio..

