# Supplementary material for: VEGF/Flk1 Mechanism is Involved in Roxarsone Promotion of Rat Endothelial Cell Growth and B16F10 Xenograft Tumor Angiogenesis
Source: Sci Rep. 2019 Nov 22;9:17417. doi: 10.1038/s41598-019-53870-3 (PMC6874592; doi:10.1038/s41598-019-53870-3)
Supplement: Supplementary file 1 — Dataset 1 [file 41598_2019_53870_MOESM1_ESM.pdf]

VEGF/Flk1 Mechanism Is Involved in Roxarsone Promotion of Rat Endothelial Cells Growth and B16F10 Xenograft Tumor Angiogenesis

Shihao Chen[1,2†], Jinge Xu[1,3†], Qianhan Wei[1], Zeting Zhao[1,2], Xin Chen[1], Hengmi Cui[2*], Yumei Zhang[1,4*]

1 College of Veterinary Medicine, Yangzhou University, Yangzhou, Jiangsu, China；

2 College of Animal Science and Technology, Yangzhou University, Yangzhou, Jiangsu, China；

3 Guizhou Animal Husbandry and Veterinary Research Institute, Guiyang, Guizhou, China；

4 Jiangsu Co-innovation Center for Prevention and Control of Important Animal Infectious Diseases and Zoonoses, Yangzhou, Jiangsu, China

[†] Shihao Chen and Jinge Xu should be considered joint first author.

[*] Hengmi Cui and Yumei Zhang should be considered joint corresponding author. Hengmi Cui, College of Animal Science and Technology, Yangzhou University, No. 12 East Wenhui Road, Yangzhou, Jiangsu 225009, China. Tel.: 86 514 87979032, Fax: 86 514 87350440, E-mail address: hmcui@yzu.edu.cn; Yumei Zhang, College of Veterinary Medicine, Yangzhou University, No. 12 East Wenhui Road, Yangzhou, Jiangsu 225009, China. Tel.: 86 514 87979044, Fax: 86 514 87972218, E-mail address: ymzhnet@sina.com.cn.

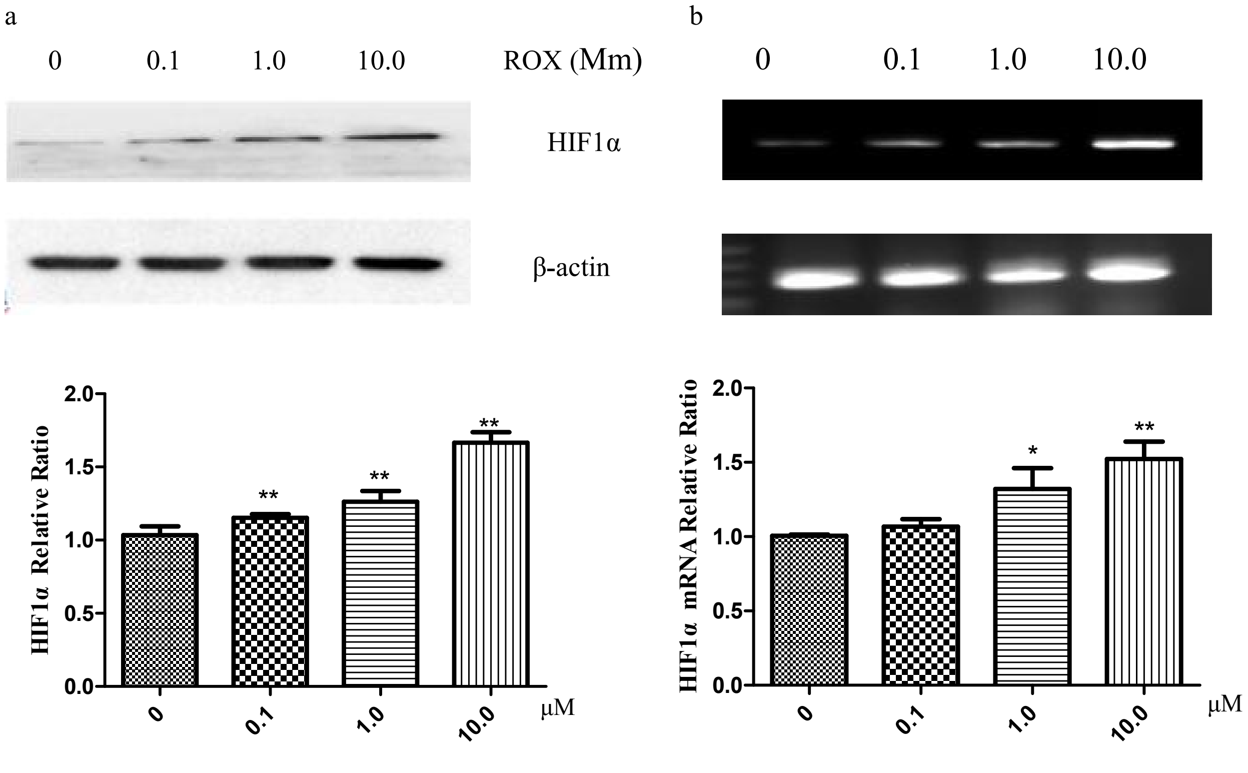

Figure S Effect of roxarsone on the expression of HIF1α in EC cultures.

a, Western blotting of total cell lysates from ECs treated with 0, 0.1, 1.0 and 10.0 μM ROX. Values are the mean ± SD of HIF1α expression standardized to β-actin expression in three independent experiments. b, RT-PCR analysis from ECs treated with 0, 0.1, 1.0 and 10.0 μM ROX. Values are the mean ± SD of HIF1α expression standardized to β-actin expression in three independent experiments. $^*P < 0.05$, $^{**}P < 0.01$ relative to PBS by ANOVA.
