# Supplementary material for: VEGF/Flk1 Mechanism is Involved in Roxarsone Promotion of Rat Endothelial Cell Growth and B16F10 Xenograft Tumor Angiogenesis
Source: Sci Rep. 2019 Nov 22;9:17417. doi: 10.1038/s41598-019-53870-3 (PMC6874592; doi:10.1038/s41598-019-53870-3)
Supplement: Supplementary file 2 — The original blots or box-plot figures [file 41598_2019_53870_MOESM2_ESM.pdf]

VEGF/Flk1 Mechanism Is Involved in Roxarsone Promotion of Rat Endothelial Cells Growth and B16F10 Xenograft Tumor Angiogenesis

Shihao Chen[1,2†], Jinge Xu[1,3†], Qianhan Wei[1], Zeting Zhao[1,2], Xin Chen[1], Hengmi Cui[2*], Yumei Zhang[1,4*]

1 College of Veterinary Medicine, Yangzhou University, Yangzhou, Jiangsu, China；

2 College of Animal Science and Technology, Yangzhou University, Yangzhou, Jiangsu, China；

3 Guizhou Animal Husbandry and Veterinary Research Institute, Guiyang, Guizhou, China；

4 Jiangsu Co-innovation Center for Prevention and Control of Important Animal Infectious Diseases and Zoonoses, Yangzhou, Jiangsu, China

[†] Shihao Chen and Jinge Xu should be considered joint first author.

[*] Hengmi Cui and Yumei Zhang should be considered joint corresponding author. Hengmi Cui, College of Animal Science and Technology, Yangzhou University, No. 12 East Wenhui Road, Yangzhou, Jiangsu 225009, China. Tel.: 86 514 87979032, Fax: 86 514 87350440, E-mail address: hmcui@yzu.edu.cn; Yumei Zhang, College of Veterinary Medicine, Yangzhou University, No. 12 East Wenhui Road, Yangzhou, Jiangsu 225009, China. Tel.: 86 514 87979044, Fax: 86 514 87972218, E-mail address: ymzhnet@sina.com.cn.

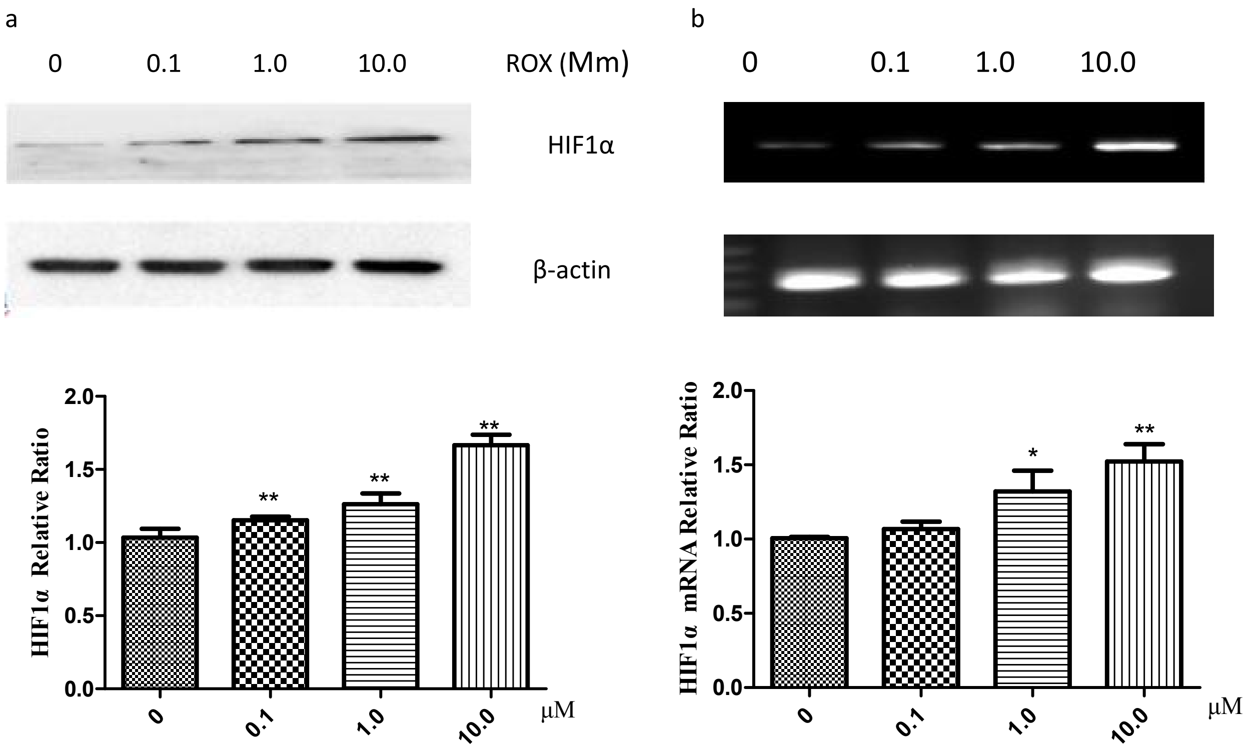

Figure S Effect of roxarsone on the expression of HIF1α in EC cultures.

a, Western blotting of total cell lysates from ECs treated with 0, 0.1, 1.0 and 10.0 μM ROX. Values are the mean ± SD of HIF1α expression standardized to β-actin expression in three independent experiments. b, RT-PCR analysis from ECs treated with 0, 0.1, 1.0 and 10.0 μM ROX. Values are the mean ± SD of HIF1α expression standardized to β-actin expression in three independent experiments. **P* < 0.05, ***P* < 0.01 relative to PBS by ANOVA.

The original WB bands of HIF1α in Fig. S

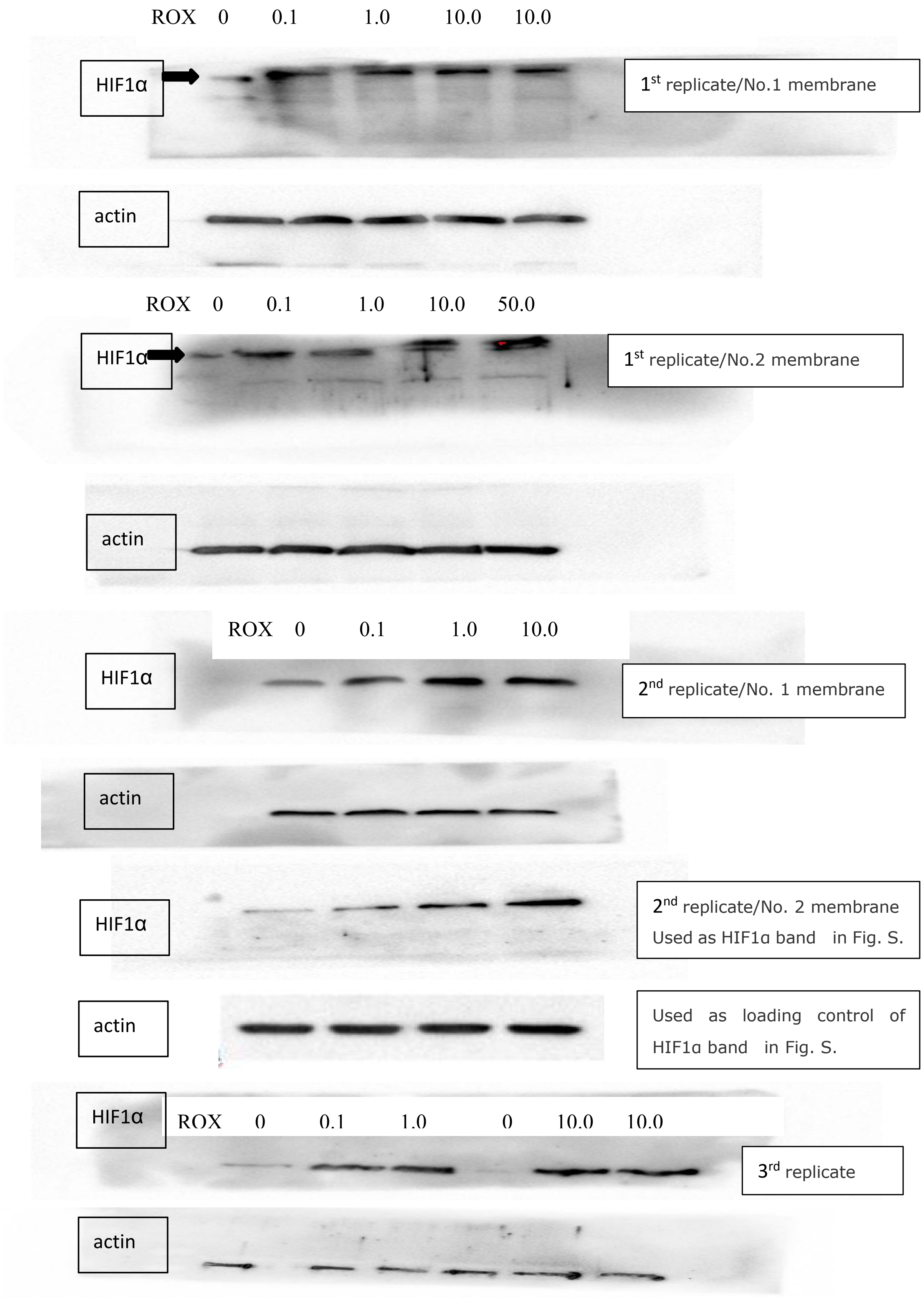

Some original images of WB or box-plot figures as below.

The WB bands for VEGF and Flk1/Flt1 used in Fig. 5

VEGF:

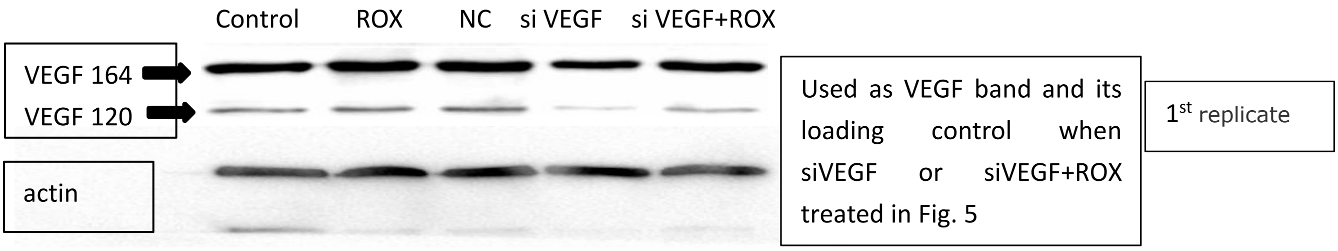

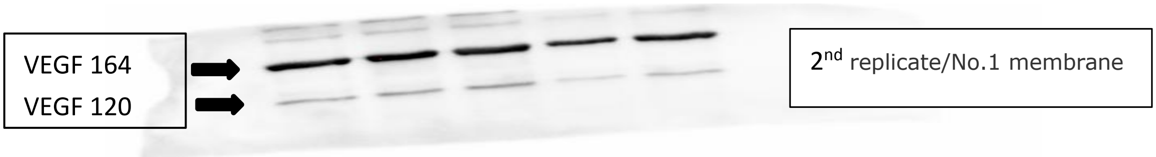

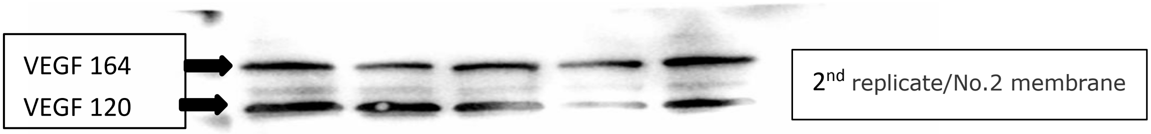

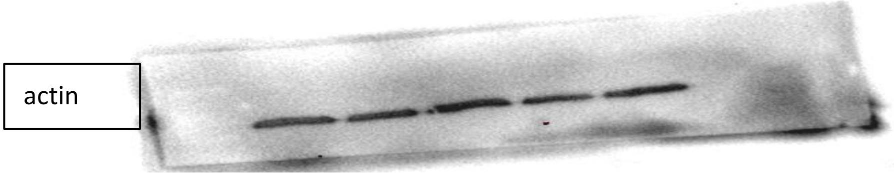

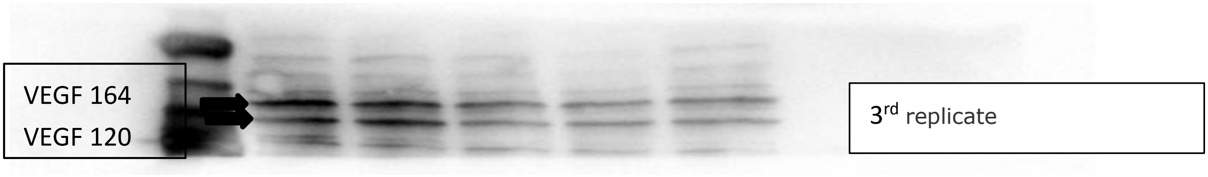

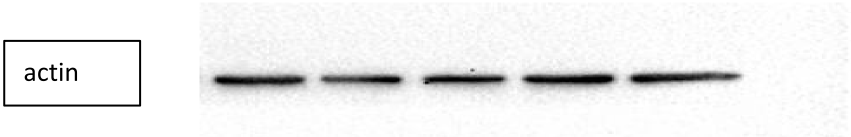

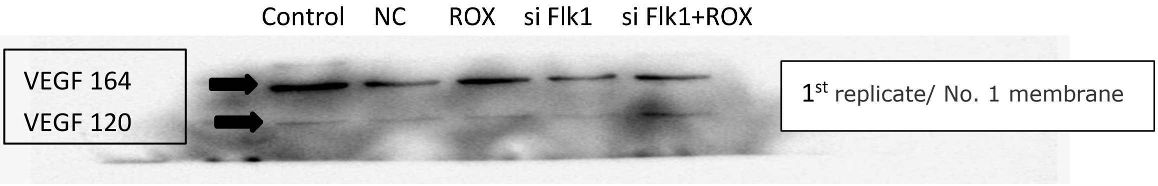

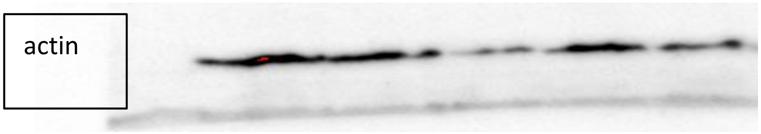
actin

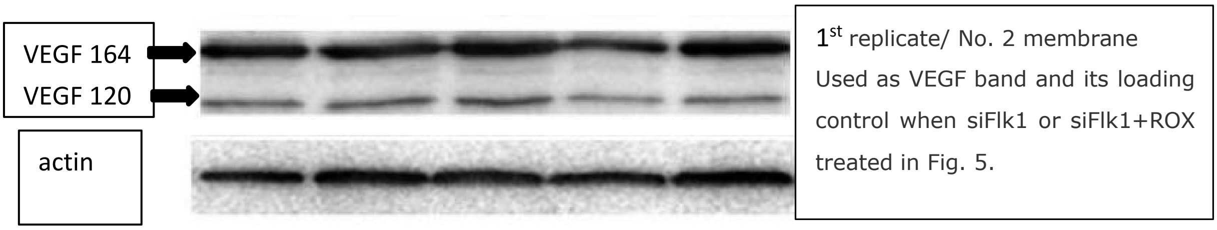
VEGF 164
VEGF 120
actin
1st replicate/ No. 2 membrane
Used as VEGF band and its loading control when siFlk1 or siFlk1+ROX treated in Fig. 5.

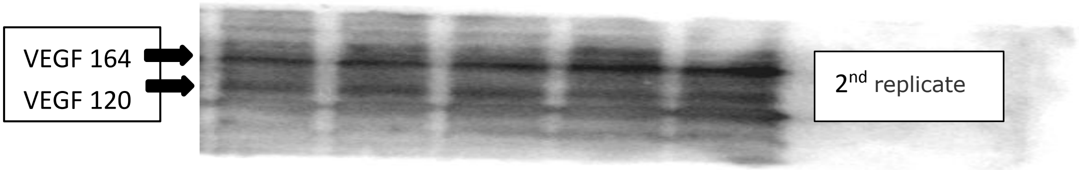
VEGF 164
VEGF 120
2nd replicate

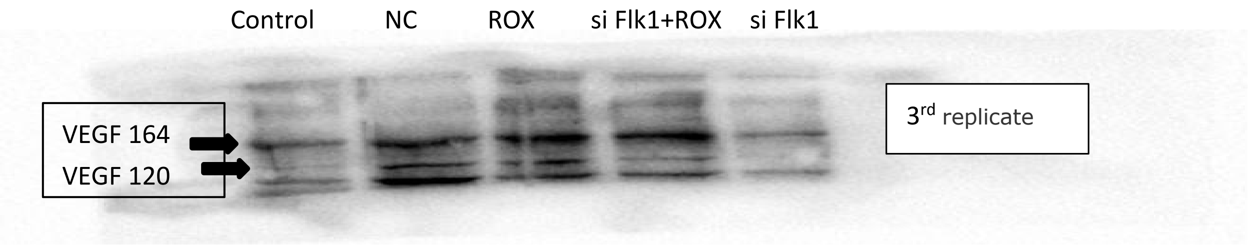
Control
NC
ROX
si Flk1+ROX
si Flk1
VEGF 164
VEGF 120
3rd replicate

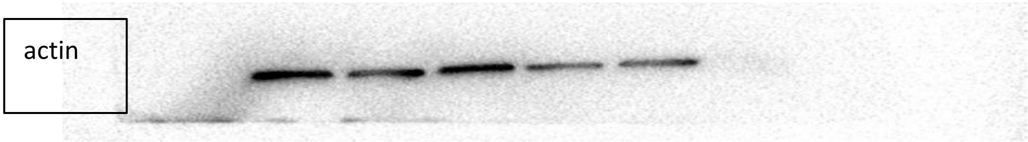
actin

Flk1:

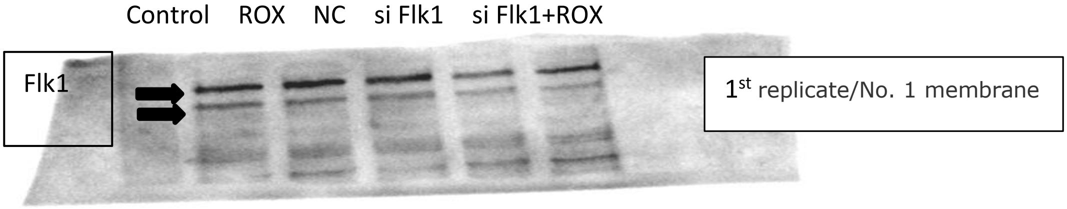
Control
ROX
NC
si Flk1
si Flk1+ROX
Flk1
1st replicate/No. 1 membrane

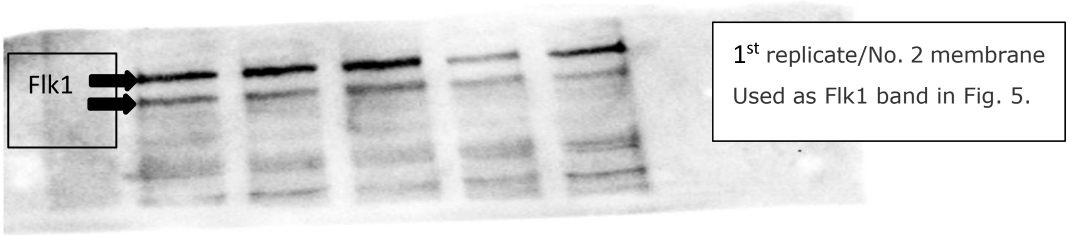
Flk1
1st replicate/No. 2 membrane
Used as Flk1 band in Fig. 5.

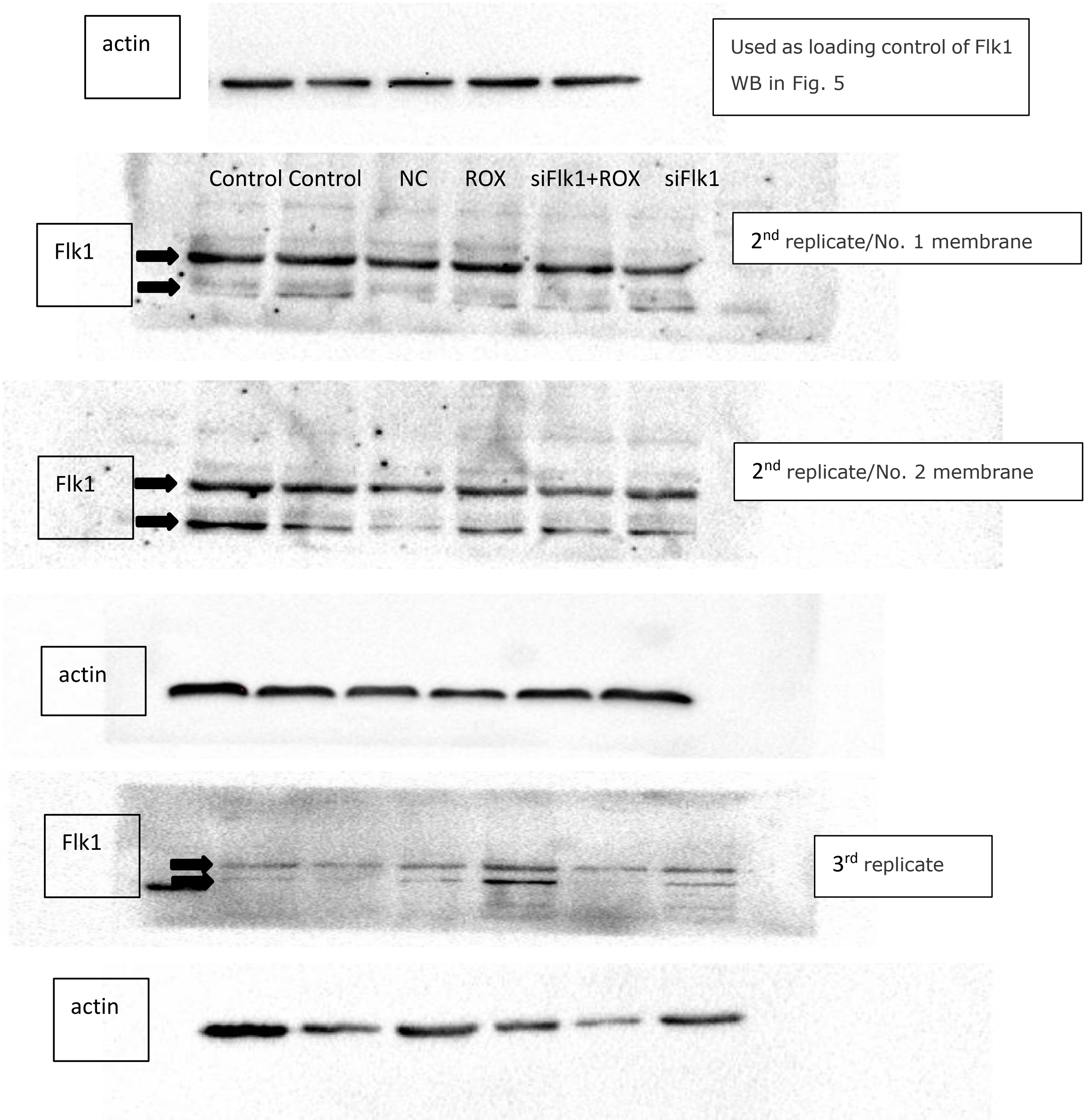
actin
Used as loading control of Flk1 WB in Fig. 5
Control Control NC ROX siFlk1+ROX siFlk1
Flk1
2nd replicate/No. 1 membrane
Flk1
2nd replicate/No. 2 membrane
actin
Flk1
3rd replicate
actin

Flt1:

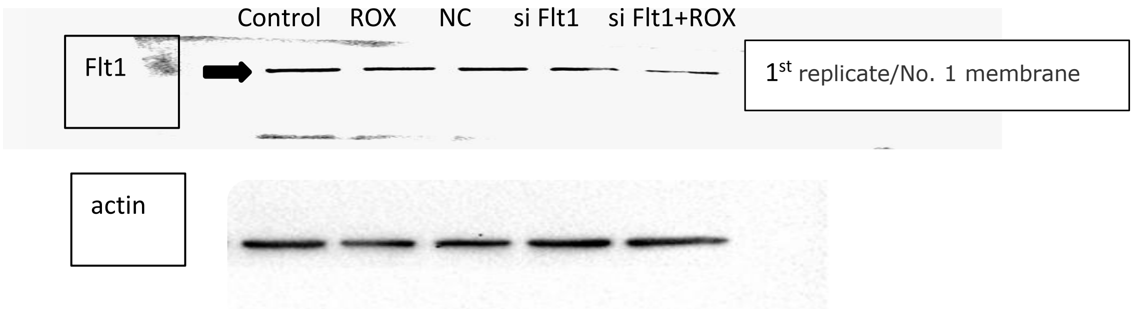
Control ROX NC si Flt1 si Flt1+ROX
Flt1
1st replicate/No. 1 membrane
actin

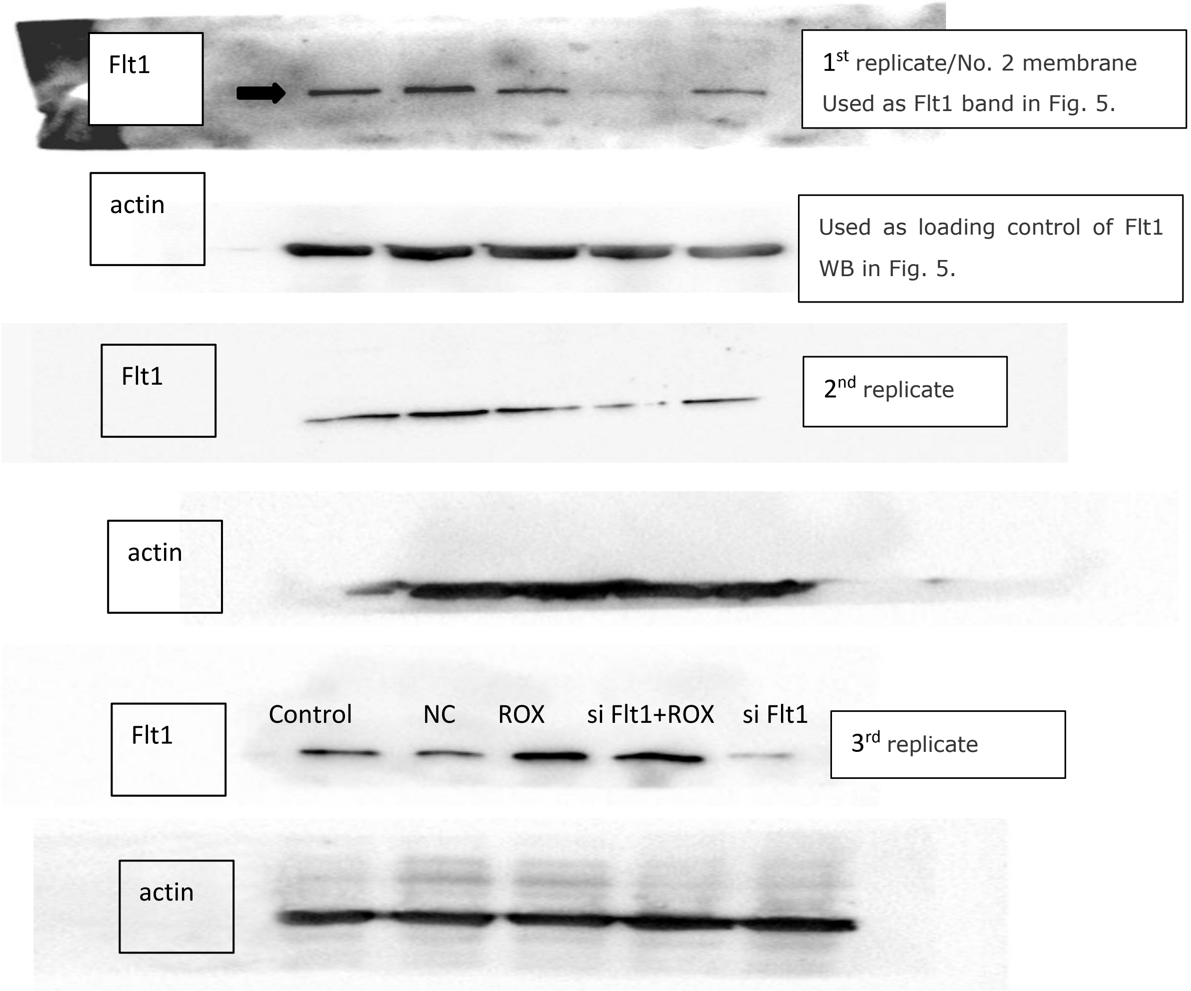

The WB bands of VEGF and Flk1 in B16F10 tumors used in Fig. 7c

VEGF

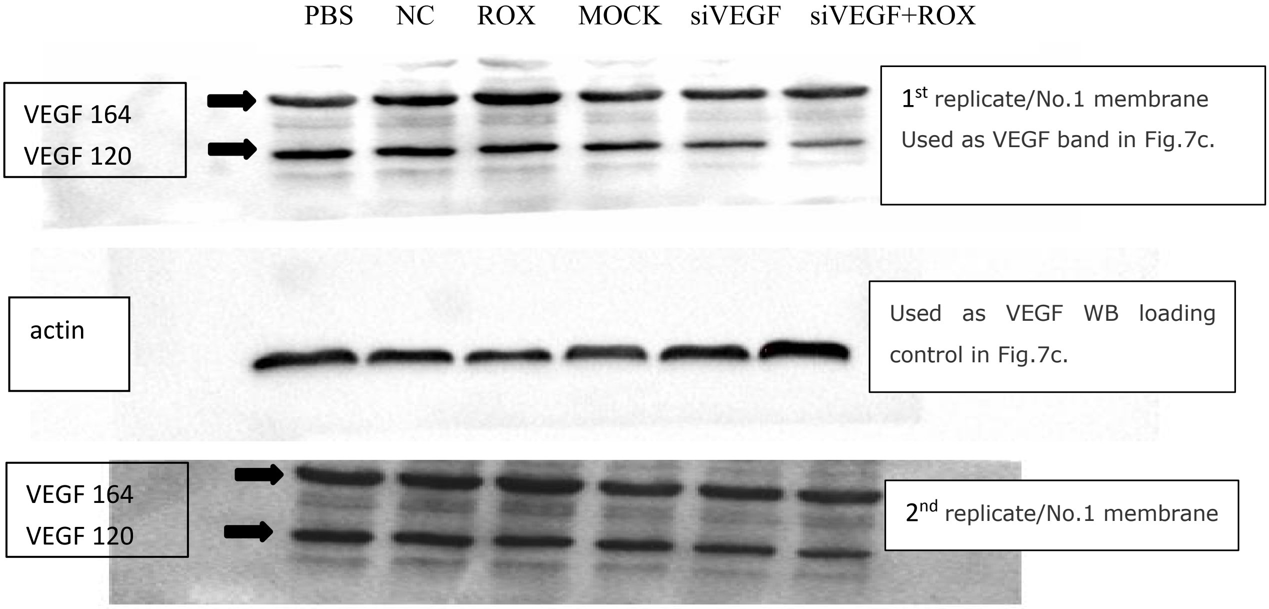

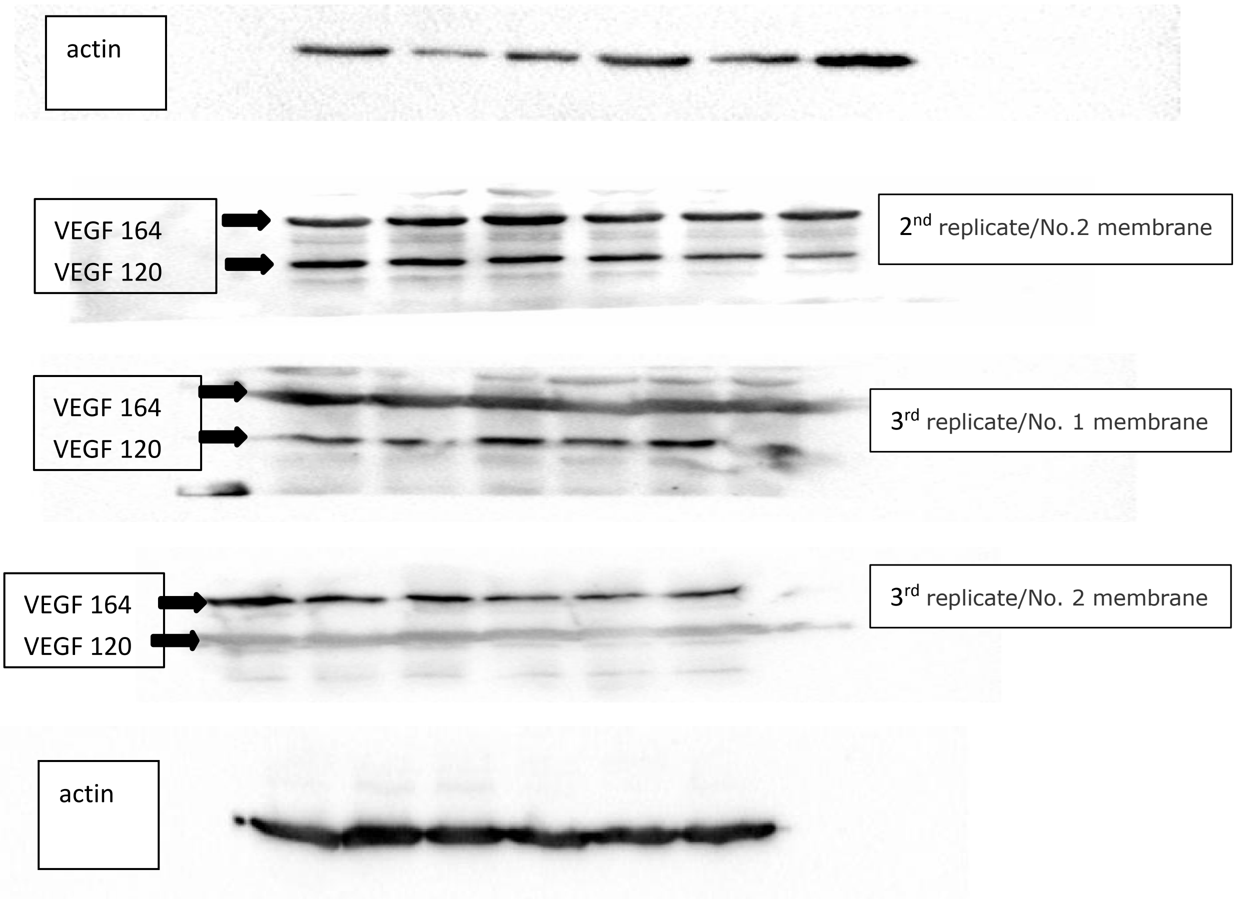
actin
VEGF 164
VEGF 120
2nd replicate/No.2 membrane
VEGF 164
VEGF 120
3rd replicate/No. 1 membrane
VEGF 164
VEGF 120
3rd replicate/No. 2 membrane
actin

Flk1:

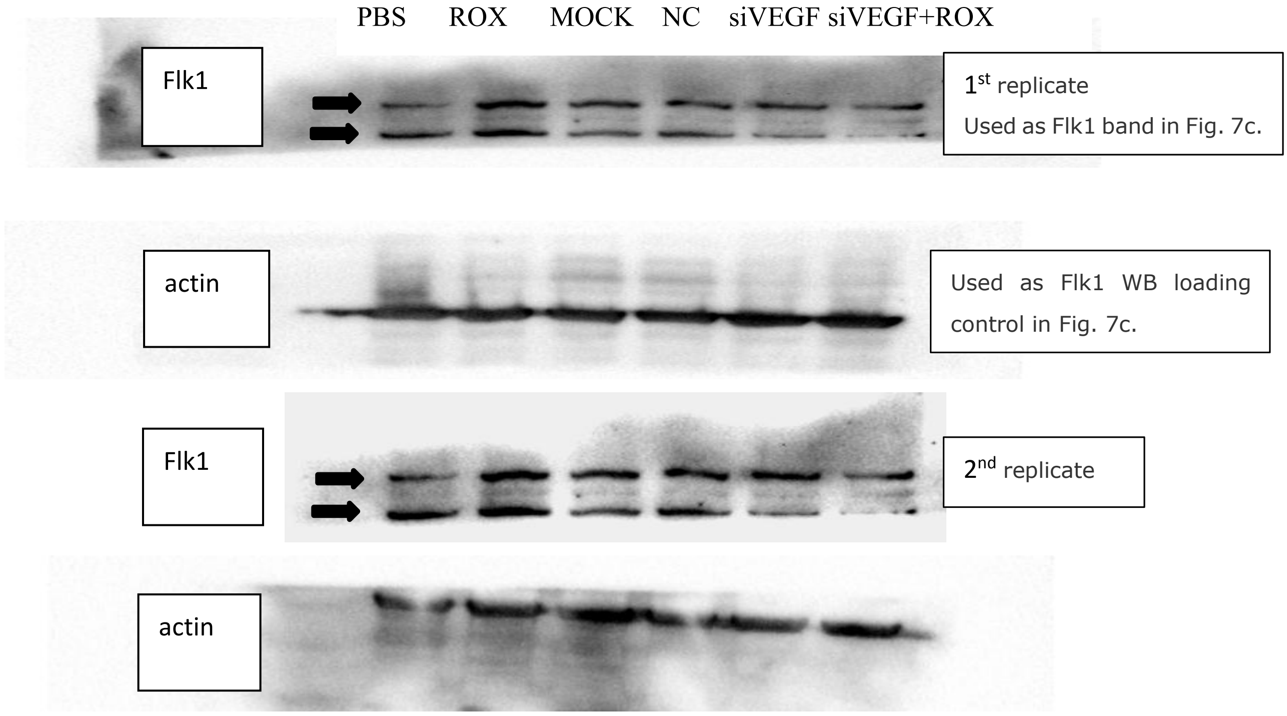
PBS ROX MOCK NC siVEGF siVEGF+ROX
Flk1
1st replicate
Used as Flk1 band in Fig. 7c.
actin
Used as Flk1 WB loading control in Fig. 7c.
Flk1
2nd replicate
actin

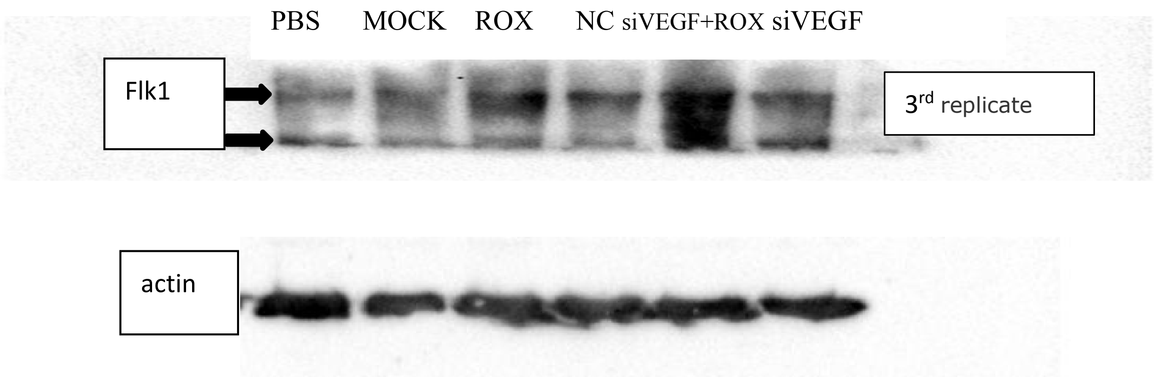

The bands of HIF by WB used in Fig.2c4

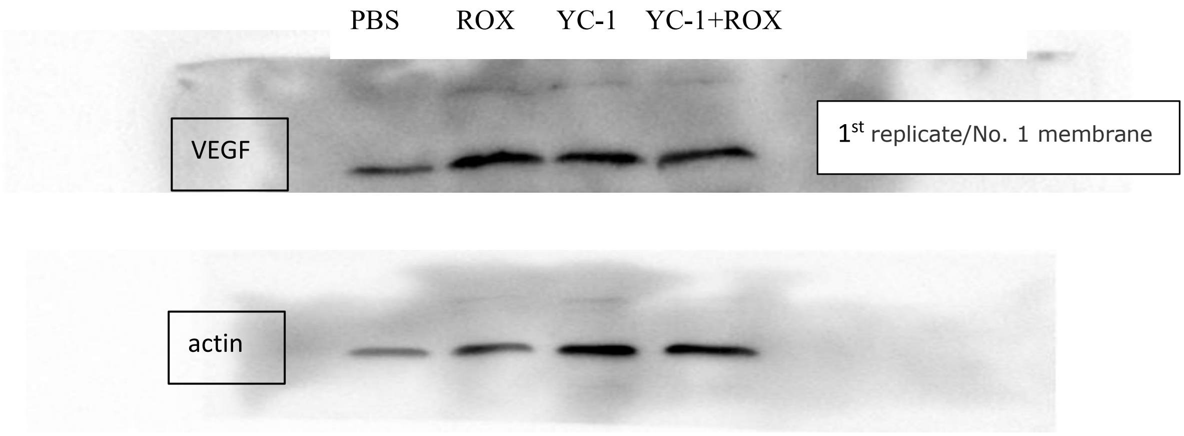

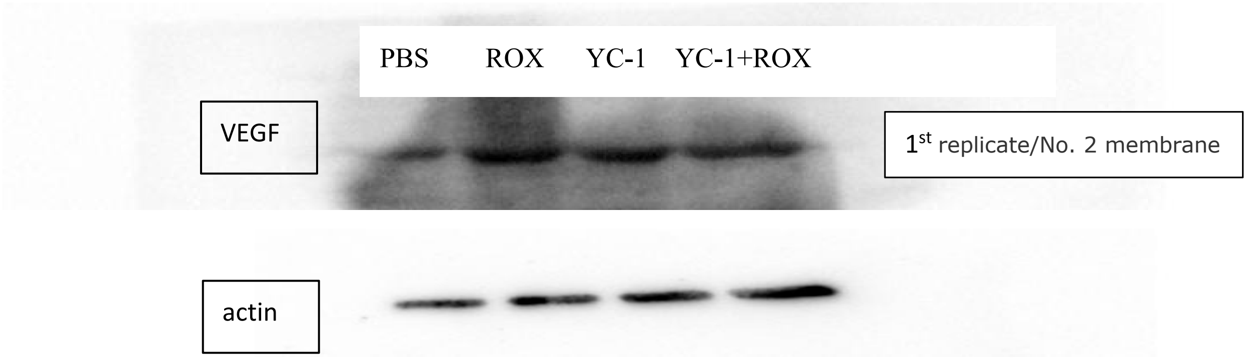

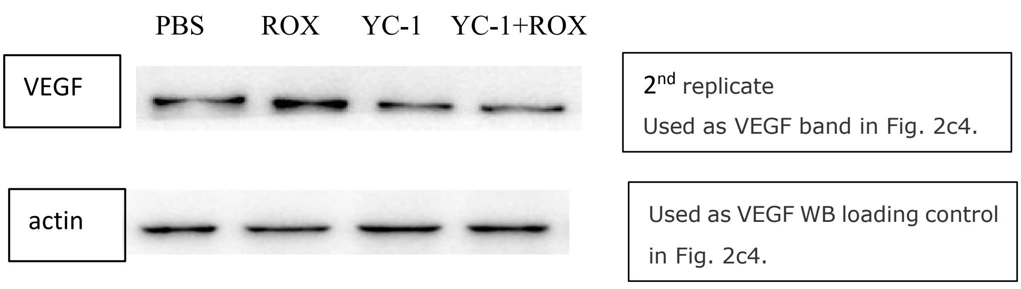

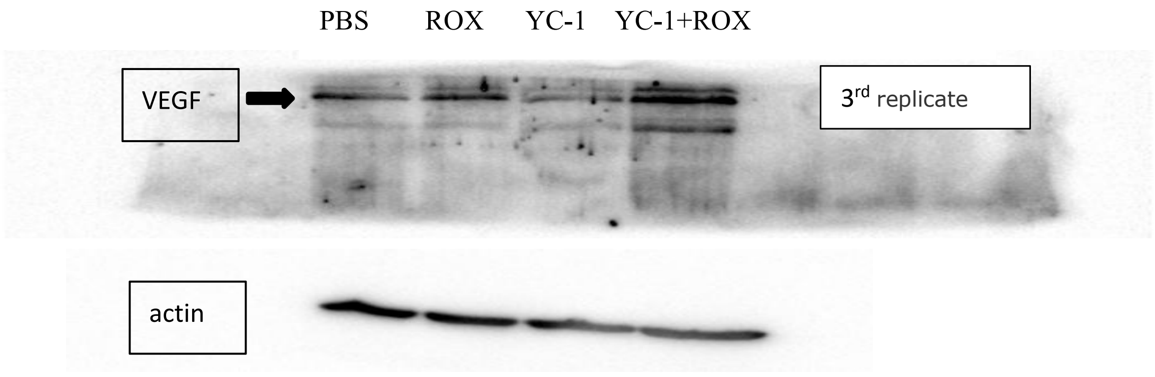

The Relative VEGF data by WB in Fig.2c3

Fig.2c3 VEGF level by WB

	PBS	ROX 1.0	ROX 1.0+anti-Flt1	ROX 1.0+anti-Flk1	anti-Flt1	anti-Flk1	anti-VEGF
1	1.0021	1.1414	1.0849	1.0364	0.9895	0.9529	0.981
2	1.0111	1.1492	1.0928	1.0411	1.0013	0.9502	0.9768
3	0.9935	1.1339	1.0771	1.0316	0.9781	0.9611	0.9921

The Relative VEGF data by WB in Fig.1d

Relative VEGF

	Control	VEGF	Rox 0.1	Rox 1.0	Rox 10.0	Rox 50.0
1	1	1.2054	1.0317	1.1414	1.0369	1.0202
2	1	1.2097	1.0437	1.145	1.0483	1.021
3	1	1.1843	1.0426	1.1377	1.0403	1.0225

Some box-plot figures as below:

Figure 3

b

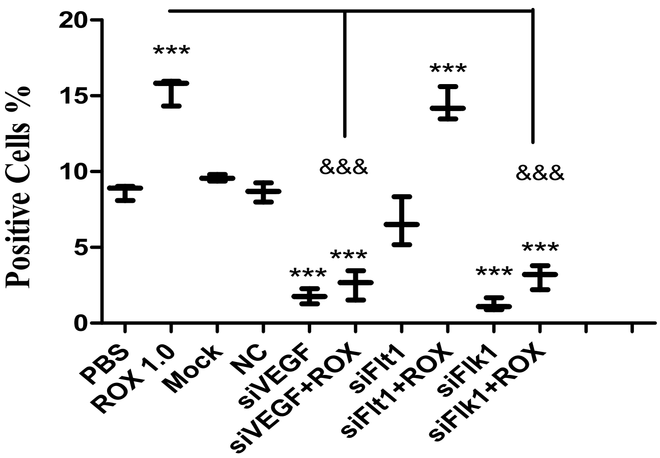

d

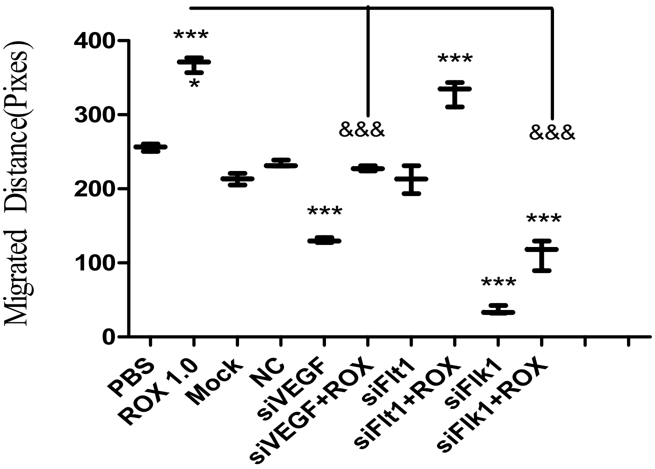

Figure 4b

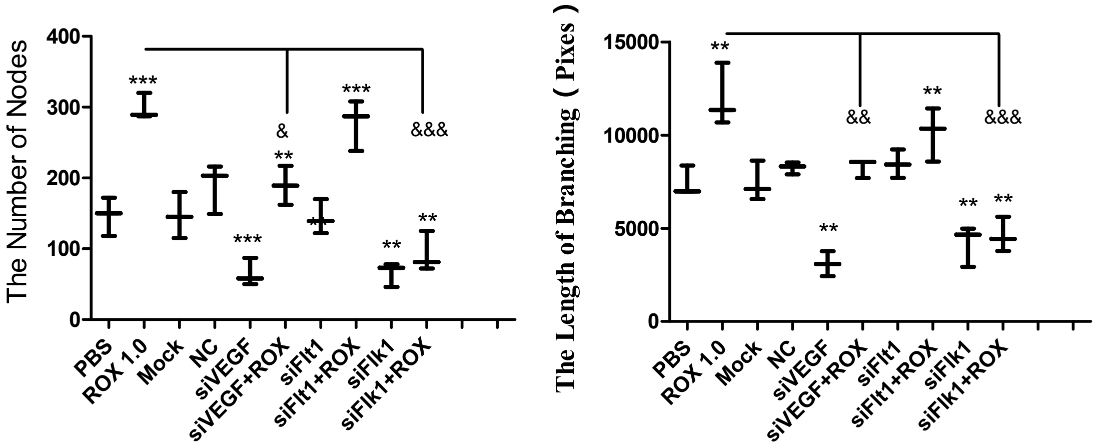

Figure 5b

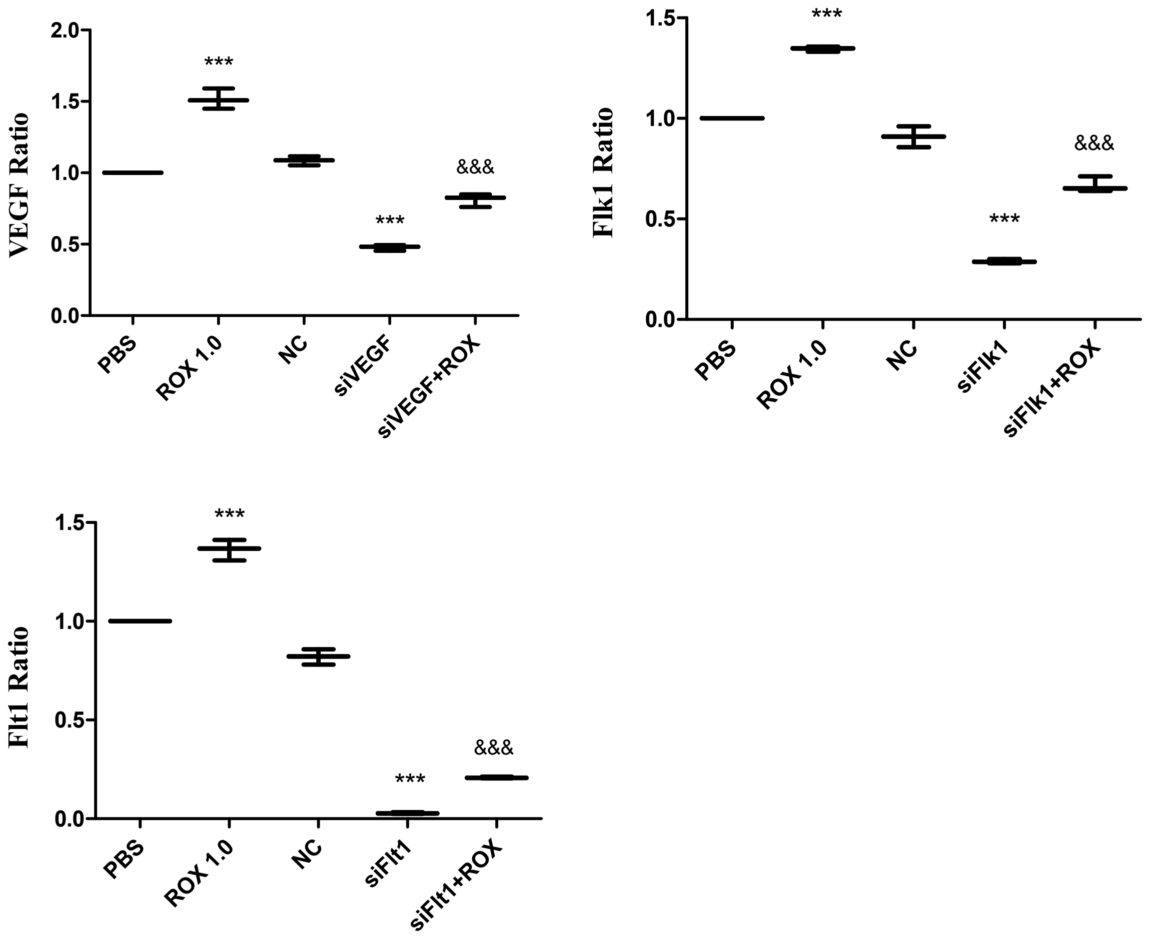

Figure 6b

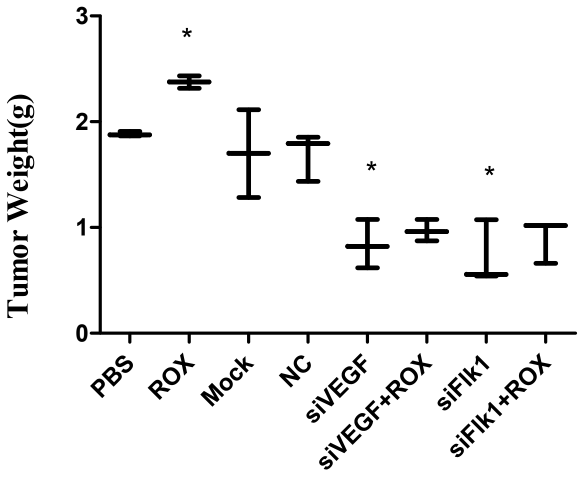

Figure 7b

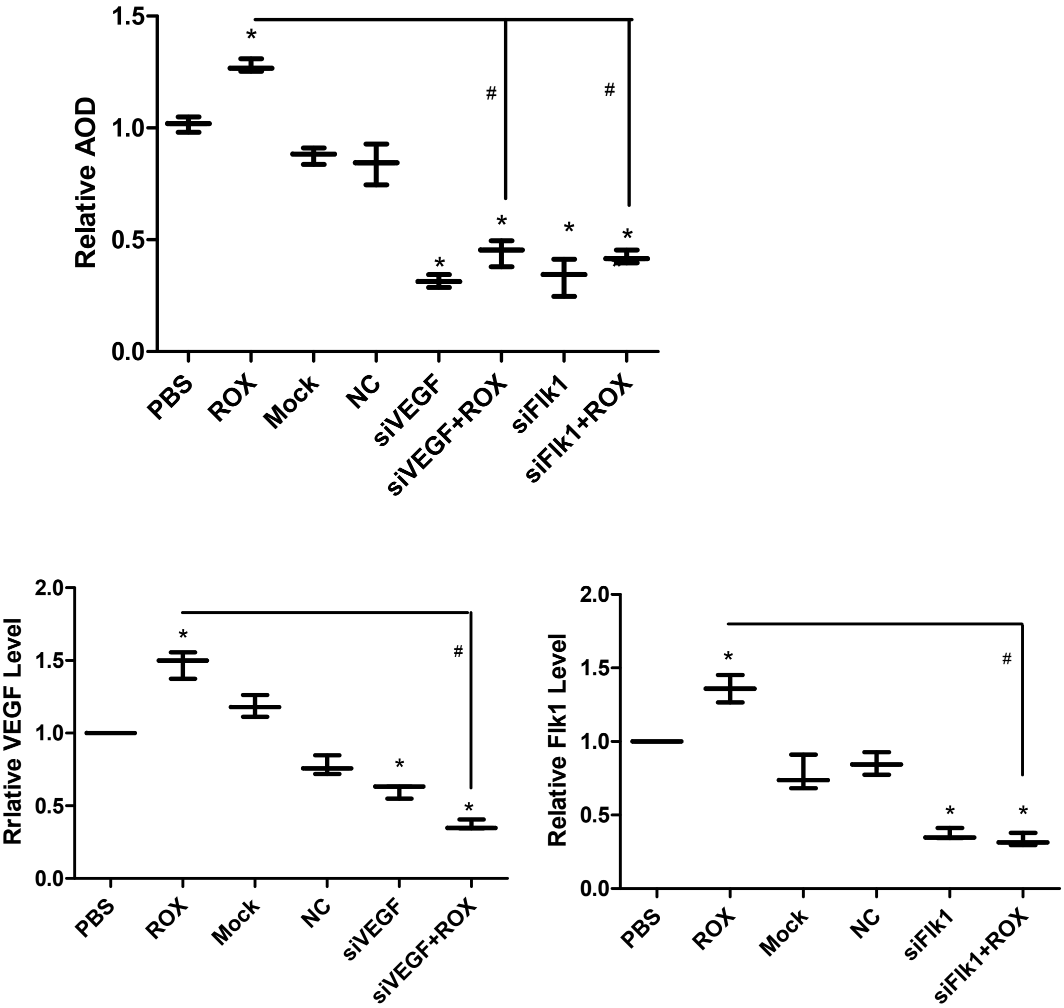

Relative AOD
1.5
1.0
0.5
0.0
PBS
ROX
Mock
NC
siVEGF
siVEGF+ROX
siFlk1
siFlk1+ROX
*
#
Rrlative VEGF Level
2.0
1.5
1.0
0.5
0.0
PBS
ROX
Mock
NC
siVEGF
siVEGF+ROX
Relative Flk1 Level
2.0
1.5
1.0
0.5
0.0
PBS
ROX
Mock
NC
siFlk1
siFlk1+ROX
